# Graphene aerogels as efficient adsorbers of water pollutants and their effect of drying methods

**DOI:** 10.1038/s41598-024-58651-1

**Published:** 2024-04-05

**Authors:** George Gorgolis, Natalia Pastra, Maria Kotsidi, George Paterakis, Nikos Koutroumanis, Christos Tsakonas, Costas Galiotis

**Affiliations:** 1 Department of Chemical Engineering, University of Patras, 26504 Patras, Greece; 2 Foundation for Research and Technology – Hellas (FORTH/ ICE-HT), Institute of Chemical Engineering Sciences, 26504 Patras, Greece

**Keywords:** Graphene aerogels, Two-dimensional materials, Water pollution, Photo-degradation, Oils adsorption, Environmental sciences, Natural hazards, Chemistry, Materials science, Nanoscience and technology

## Abstract

Environmental accidents highlight the need for the development of efficient materials that can be employed to eliminate pollutants including crude oil and its derivatives, as well as toxic organic solvents. In recent years, a wide variety of advanced materials has been investigated to assist in the purification process of environmentally compromised regions, with the principal contestants being graphene-based structures. This study describes the synthesis of graphene aerogels with two methods and determines their efficiency as adsorbents of several water pollutants. The main difference between the two synthesis routes is the use of freeze-drying in the first case, and ambient pressure drying in the latter. Raman spectroscopy, Scanning Electron Microscopy (SEM), X-ray diffraction (XRD), X-ray Photoelectron Spectroscopy (XPS) and contact angle measurements are employed here for the characterisation of the samples. The as-prepared aerogels have been found to act as photocatalysts of aqueous dye solutions like methylene blue and Orange G, while they were also evaluated as adsorbents of organic solvents (acetone, ethanol and methanol), and, oils like pump oil, castor oil, silicone oil, as well. The results presented here show that the freeze-drying approach provides materials with better adsorption efficiency for the most of the examined pollutants, however, the energy and cost-saving advantages of ambient-pressure-drying could offset the adsorption advantages of the former case.

## Introduction

Environmental disasters, such as the oil spill in Mexico gulf^1^ or, more recently, the Mauritius oil spill caused by a bulk carrier vessel^2^, are some of the ecological issues that the modern society faces frequently. These accidents highlight the need for development of efficient materials that can be employed to eliminate pollutants including crude oil and its derivatives, as well as toxic organic solvents like volatile organic compounds (VOCs). VOCs can be found in drinking water as discharge from industries or from disinfection byproducts^3^. This is a significant health concern, since VOCs have been recognised as potential carcinogens. Xylenes, benzene, ethylbenzene, trichloroethylene and tetrachloroethylene are mainly detected in the air, while formaldehyde and dichloromethane are found in water^4,5^. To tackle these problems, several novel materials have been proposed for the purification processes of such contaminated areas with graphene-related materials having an important role^6–8^.

The synthesis of 3D graphene-based materials derives from the need to exploit the properties of graphene at a larger scale^9^ and via facile preparation methods. Graphene aerogels (GAs) have been investigated for their significantly low density^1^, high electrical conductivity ^10^, organic dye/ pollutant removal^11^ and other applications^1,9, 12–17^. Ambient pressure drying (APD) is an alternative gel drying method for the final step on aerogel synthesis. Supercritical drying and freeze-drying are two methods that are significantly energy consuming and costly, while APD is quite simple in implementation and viable even at large-scale production. It is based on the application of subcritical pressure and simple liquid evaporation^18^. It takes place at ambient pressure and needs only a common refrigerator and a common oven. On the other hand, the freeze-drying method requires a specific apparatus while due to the applied conditions (very low temperature and pressure), the solvent sublimates from the solid to the gas state^19^.

As a response to the major issue of water purification/filtration, several sorbent materials like microporous nanocomposites, and carbon nanotubes^20^ have been till now examined for a lot of separation and purification procedures. For the dyes adsorption study like methylene blue (MB), except GAs, other kind of aerogels have been exploited as silica aerogels^21,22^, cellulose^23^ and nanocellulose aerogels^24^. All these materials exhibit high surface area, compressive modulus and recyclability and thus, can be considered as reusable adsorbents in wastewater treatment.

Graphene-related materials (GRMs) can be used for the adsorption of oils and organic solvents, mainly GO and rGO due to the lower cost of the composite structures, as well as the larger specific surface area in comparison to the pristine graphene. GRMs demonstrate much higher adsorption of oils and organic solvents than conventional adsorbents, taking into consideration the abundant ways of surface modification that ameliorate the properties of the graphene adsorbents. Therefore, the need for developing functionalised graphene-based porous materials with high adsorption capacity and chemical inertness for a broad range of organic solvents and oils is more compelling than ever.

the present study, the synthesis of graphene aerogels both with freeze-drying and ambient pressure drying, and, the comparison of their efficiency as adsorbents of water pollutants, is described. The conventional method for the drying (freeze-drying) is compared to a greener approach (ambient-pressure drying), under a common target that is the pollution of water in the form of volatile organic compounds, organic solvents and oils.

## Materials and methods

Graphene aerogels were prepared according to the information given by Hong et al.^25^ and Yang et al.^18^. Both methods are based on the chemical reduction of graphene oxide as the first step and the formation of the aerogel as the second, providing a more cost-effective solution and resulting in a lattice structure similar to graphene. The main difference between the two synthesis routes is the use of a freeze-dryer in the first case, and ambient pressure drying process in the latter. Even though acquiring a stable 3D structure from a hydrogel is most commonly achieved with freeze-drying, the alternative of ambient pressure drying suggests significantly less energy consumption and a more facile approach that could potentially be scaled-up. In the SI file, the preparation of graphene oxide and graphene aerogels with both freeze-drying and ambient-pressure drying are analytically described. The surface modification route for the aerogels obtained with FD is also explained.

### Characterisation

For the characterisation of the specimens, SEM photos were taken using a LEO SUPRA 35 VP, while Raman spectra of the specimens were recorded using an InVia Reflex (Renishaw, UK) MicroRaman equipment using a 632 nm laser excitation. The XPS measurements were carried out in an ultra-high vacuum system (UHV), which consists of a fast entry specimen assembly, a sample preparation, and an analysis chamber equipped with a dual anode (Al/Mg) X-ray gun and an LH10 electron analyzer. For the X-Ray diffraction measurements, a Bruker D8 Advance model was used, while the contact angle experiments were performed with a KRÜSS DSA 100 contact angle meter with the sessile drop method. Measurements were performed three times, and the average values of contact angles were calculated. The electrical resistance (conductivity) measurements were performed by using a Keithley 2420 source meter unit (SMU) in a four-probe configuration. Specific surface area and porosity measurements were performed by obtaining the volumetric nitrogen adsorption at 78 K, using an “autosorb iQ Model 7” gas sorption system and high purity gases (> 99.999%). The specific surface area was found following the multi-point BET equation. The pore volume was calculated from the adsorbed nitrogen at 0.90 relative pressure. The pore size distribution was determined with the Barrett-Joyner-Halenda (BJH) model. Zeta potentials were measured on a particle size and zeta potential analyser (Nano Zeta sizer-Malvern) using deionised water as solvent. The absorbance spectra of the examined solutions with and without the aerogels were acquired using a HP 8452A Diode Array UV–VIS spectrophotometer, working in a λ range of 190–830 nm (with 2 nm of resolution).

### Experiments of photo-degradation of dyes and adsorption

The as-synthesised materials were investigated for their adsorption capacity for water pollutants. In particular, aqueous dye solutions were evaluated with and without the presence of GAs, and, with and without light, with the assistance of UV–Visible spectroscopy. The aerogels indeed exhibit some adsorbing ability, however they contribute mostly as photocatalysts of the dyed solutions. Afterwards, the GAs were evaluated as adsorbents of organic solvents, namely acetone, ethanol and methanol, and VOCs like formaldehyde and dichloromethane. After being soaked, their weight gain was determined to conclude the maximum adsorption. Based on the theoretical hydrophobicity of the material, some samples underwent soaking in pump oil, castor oil and silicone oil to support the claim that they could be applied in separation processes of oil from aqueous solutions. Finally, GAs synthesised with the freeze-drying method, incurred surface modification from a fluorine silane coupling agent^25^.For this study, the batch method was used. Vessels with similar size filled with the examined solvent, were placed inside desiccators^15^, under the same conditions of temperature and pressure^12^.

## Results and discussion

### Characterisation of the synthesised graphene aerogels

**Figure 1:**
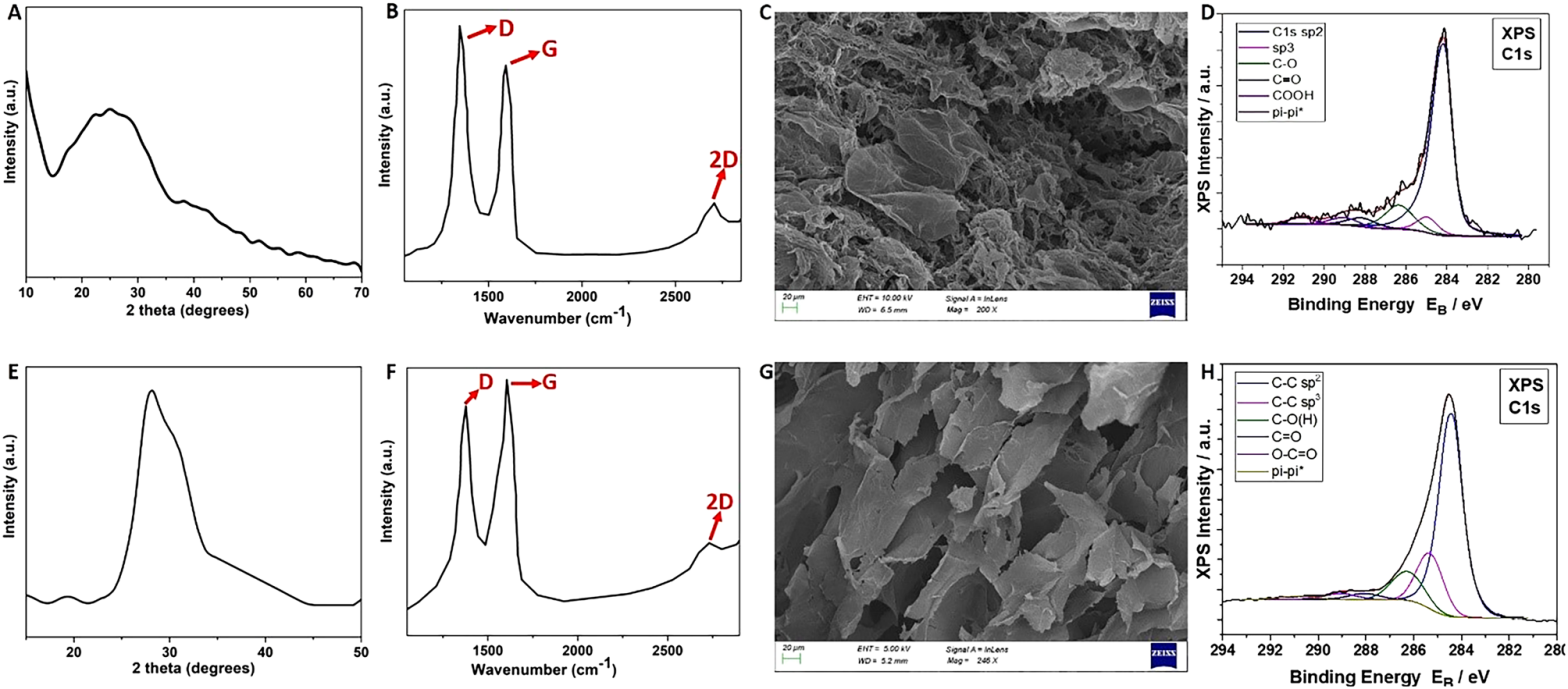
Upper row—graphene aerogel prepared with freeze-drying. (**A**) XRD plot, B) Raman spectrum for incident wavelength of 632 nm, (**C**) SEM image with a scale bar of 20 μm, (**D**) Deconvoluted C1 peak from the XPS survey scan. Lower row—graphene aerogel prepared with ambient-pressure drying. (**E**–**H**) depict correspondingly the results from the same techniques used in the upper row.

Densities which lie in the range of 14–25 mg/cm^3^ have been calculated for the specimens prepared with both methods. An XRD analysis was performed for both the freeze-dried (FD) and the ambient-pressure dried (APD) graphene aerogels so as to determine the crystallinity of the materials, and it is shown correspondingly in Fig. 1A,E. In the first case, the XRD plots showed a broad peak at 2θ = 26° which corresponds to the (002) plane of graphite structure. In the case of APD aerogels, the peak is observed at approximately 28°, verifying the reduction of graphene oxide. From the Raman spectrum of Fig. 1B, it can be deduced that the presence of D and G peaks of the sample are in agreement with literature, which suggests that these are characteristic of graphene oxide and reduced graphene oxide. The ID/IG ratio that quantifies the defects in the lattice and the conjugation disruption was calculated at 1.19, which compares well to the value of 1.27 calculated by Hong et al.^25^. In comparison, the Raman spectrum of the GAs synthesised by the APD method also exhibits the G and D characteristic peaks for graphene oxide and rGO (Fig. 1F) which appear at 1605 and 1377 cm^-1^ wavenumbers, respectively. These peaks confirm that the main identifying bonds of the material are the sp^2^ hybridized bonds. Moreover, there is a distinct 2D peak, as previously, while the ID/IG ratio in this case is calculated at 0.89.

The SEM analysis of the samples gave information on their morphology and their pore sizes. The FD graphene aerogels reveal pores in the order of magnitude of micrometer and some nanometers (Fig. 1C). It is evident from the obtained figures that layers of reduced graphene oxide with ripples and wrinkles are present. It is evident that the majority of the pores are of some microns, thus confirming that the 3D material is mainly macroporous, similar to^25^. Accordingly, another SEM observation was carried out so as to establish the type of porosity of APD graphene aerogels (Fig. 1G). The survey suggests that most of the pores are also micro-sized and the material can be characterised as macroporous. This is in agreement with literature^18^, where all the prepared aerogels exhibit a macro-porosity in their structure.

The surface analysis of FD graphene aerogels indicated the presence of C, O and P atoms. Figure 1D below represents the convoluted XPS C1 peak that correspond to the sp^2^ hybridised C–C bonds and is analysed into: Binding Energies (BE) 284.4 ± 0.1 eV assigned to C–C sp^2^ hybridization, 285.2 ± 0.1 eV assigned to C–C sp^3^ hybridization, 286.3 ± 0.1 eV assigned to epoxides and/or hydroxides (C–O(H)), BE = 288.1 ± 0.1 eV assigned to carbonyls (C = O), 289.0 ± 0.1 eV assigned to carboxyls (–COOH), 291.1 ± 0.1 eV due to pi-pi* transition loss peak. From the peak intensities of C1s, O1s and P2p of Supplementary Fig. 5, the % relative atomic ratio is extracted and the results are shown in Supplementary Table 1. The ratio is C:O = 10.8, suggesting successful reduction of GO to rGO. The analysis was repeated for a sample prepared with APD method in order to determine the level of graphene oxide reduction and to detect the atomic ratios of the material. The deconvoluted C1s peak (Fig. 1H) was analysed into: C–C with sp^2^ and sp^3^ configuration at binding energies 284.4 and 285.3 ± 0.1 eV, respectively, C-O hydroxides or/and epoxides at 286.3 ± 0.1 eV, carbonyls C = O at 287.8 ± 0.1 eV, carboxyls, COOH, at 289.3 ± 0.1 eV, pi–pi* transition loss peak at ~ 291 eV. The % relative atomic concentration of carbon and oxygen atoms are determined from the total areas of C1s and O1s, and it was found as 85.63 ± 0.04 for C and 14.37 ± 0.04 for O. The C:O ratio in this case is 5.95.For comparison, the C:O ratios of XPS surveys for graphene aerogels from various reports in literature are presented in Supplementary Table S2.

The specific surface area (SSA) of the aerogels was found to be equal to 27 m^2^/g for the FD and 18m^2^/g for the APD, relatively low for GAs^25^ but obtained in similar studies for such materials^26^. Certainly, the majority of the existing pores within the aerogels is in the range of micrometers, as already shown in the SEM images. Based on the shape of the FD aerogel’s nitrogen adsorption–desorption isotherm (Supplementary Fig. 9), the material shows a Type II isotherm with a Type H3 hysteresis loop, according to IUPAC classification. The pore network is mainly consisted of macropores which are not completely filled with pore condensates. A similar behaviour was observed also for the APD GA. Additionally, from the pore size distribution of Supplementary Fig. 9, it can be deduced that the studied aerogel is characterised by both meso-porosity (between 2 and 50 nm) and macro-porosity (> 50 nm). In the Supplementary Table 3, the pore volume and pore surface area for the corresponding diameters are shown.

The hydrophobicity of the GAs was determined by contact angle analysis. GAs samples were prepared with FD method and some of them had been functionalised according to the experimental process described in the SI file. The aerogel which was not functionalised exhibited an average *θ*_*c*_ of 90.9°, while the functionalised aerogel had an average *θ*_*c*_ of 118.3°. From the corresponding Supplementary Fig. 10, it is evident that the hydrophobicity of the functionalised graphene aerogel has been noticeably increased, while from literature, it is known that a GO aerogel shows a water contact angle equal to 65–66°^26^. The electrical conductivity of the GAs has been also measured and found equal to 40.40 S/m for the FD method and 14.67 S/m for the APD method. The relatively high conductivity of the FD GAs indicates the effective reduction of the GO which is in agreement with the results from the XPS measurements. This value is close to the highest reported conductivities in the range of 50–87 S/m for GAs with similar densities. The difference of the conductivity between the two methods is attributed to the extent of the reduction of the rGO. At any rate, the electrical conductivity is considered reasonably high for both cases.

### Photo-degradation of dyes

Methylene blue (MB) is considered as a model pollutant due to its role as an absorbate in solids, and thus its behaviour is investigated for the removal of organic pollutants from aqueous solutions. MB was used in a series of experiments in order to study the adsorbing capacity of the synthesised GAs. Firstly, aqueous solutions of MB were synthesised and afterwards their absorbance with and without light, and, with and without the presence of GAs was examined. MB as a cationic dye, exhibits two major absorbance peaks at 293, due to π-π* transition, and at 664, due to n-π* transition. The obtained spectrum of Supplementary Fig. 12 is in accordance with spectra derived from literature. Furthermore, the characteristic shoulder at approximately 610 nm is also apparent. Supplementary Table S4 shows the composition details of the aqueous MB solutions along with the corresponding absorbance peaks of the UV–Vis spectra.

To determine the adsorbing capacity of the GAs, MB aqueous solutions were tested both with and without exposure to ambient light. In the first case, a 2.1% w/w MB aqueous solution was prepared. In two beakers, equal parts of the solution and two FD aerogels were inserted, while the solution remained exposed to ambient light for the duration of the experiment. Every ten minutes the spectrum of each MB solution was obtained with the UV–Vis spectrophotometer for totally 2 h and 50 min. In addition to FD GAs, the APD GAs were tested as well. For both FD and APD, the same quantity of aerogel was used, equal to 20 mg. The same aqueous solution of MB was used in order to obtain comparable results. The spectrum of the solution was obtained every 10 min for the first hour and, then, every 15 min. The overall duration of the experiment was 2 h and 30 min.

**Figure 2:**
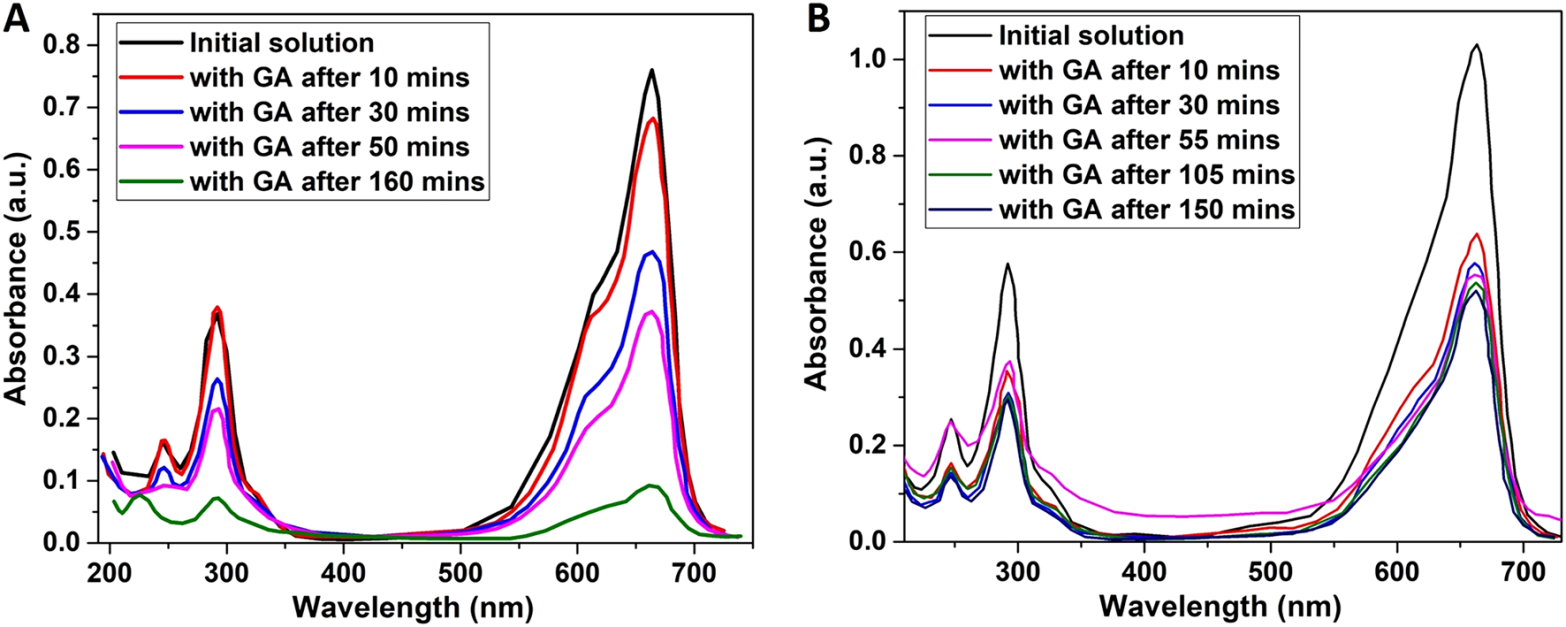
(**A**) UV–Vis’s spectra of two MB aqueous solutions each containing one graphene aerogel with light exposure, and, (**B**) UV–Vis’s spectra of a MB aqueous solution containing a graphene aerogel (APD synthesis method) with light exposure.

In both cases, there is a significant decrease of the maximum absorbance peak at the UV–Vis spectra of MB. The GAs adsorb part of the pollutant, but also assist and accelerate its photolysis, thus acting as photocatalysts. To compare the difference in the adsorption and photocatalysis phenomena of a MB solution without the presence of light,an initial solution of 0.0007 mg/mL concentration was prepared and was sealed from light exposure. A FD graphene aerogel was soaked in the solution and UV–Vis’s spectra were obtained every 10 min for 2 h and 40 min (Supplementary Figs. 13–14). Comparing Fig. 2 to Supplementary Fig. 13, it is evident that the presence of light affects strongly the methylene blue adsorption from the GA. To eliminate any doubt of the catalytic capacity of the aerogels, the UV–Vis spectrum of a methylene blue aqueous solution was obtained without light exposure and without the addition of a GA. The experiment took place for 1 h and 50 min, and, measurements were taken every 10 min. It is evident from Suppl. Figure 15 that the dye is hardly photodegraded in the presence of light, while the degradation is significantly accelerated with the use of the prepared GA.

From Fig. 2, it can be deduced that the FD GAs demonstrate a superior performance as photocatalytic materials than those prepared with APD. For the photocatalytic degradation of MB, the constant rate has been calculated equal to 0.013276 min^−1^ and 0.003020 min^-1^, respectively for FD and APD. Compared to other reported graphene and carbon-based aerogels which present corresponding rates equal to 0.0024 – 0.0545 min^−1^^27–31^but lie mostly in the range of 0.010 – 0.030 min^−1^, the FD aerogels can be considered as very competitive. While the same cannot be argued for the APD ones. It can be observed that for FD (Fig. 2A), a decrease of the absorbance intensity is recorded from 0.413 to 0.073 a.u. (absorbance units), and from 0.751 to 0.091 a.u., for 292 nm and 664 nm respectively. This implies a decrease of 82% and 88% respectively for the two peaks. Correspondingly for APD (Fig. 2B), the absorbance intensity decreases from 0.580 to 0.302 a.u. for 292 nm, and, from 1.037 to 0.542 a.u. for 664 nm. The superior photocatalytic efficiency of MB for the FD GAs versus the APD GAs can be attributed to the chemical doping of the former with phosphorus (P) originating from hypophosphorous acid (H_3_PO_2_), in combination with lower (about the half) rates of GO reduction for the latter.. Chemical doping with P has been reported as an effective strategy to tailor the electronic properties of graphene, something that is essential for the improved photocatalytic activity. Supplementary Fig. 16 demonstrates the ability of the FD GAs to purify efficiently the aqueous MB solutions after two days. The biggest batch of dye adsorption studied was a MB aqueous solution of 50 ml, from a FD aerogel of 100 mg weight, while the adsorption of MB in dark is equal to 8.5 mg/g. This value is much lower than reported high adsorption capacities onto various graphene-based aerogel adsorbents like 1023.9 mg/g by Yan et al., 652.99 mg/g by Liu et al.^27^ and 578 mg/g by Chen et al. ^32^. Furthermore, the recyclability of the GAs prepared by both methods was achieved following the procedure described in^26^ using HCl (10%, v/v) and ethanol solutions (50%, v/v). In Supplementary Table 5, the degradation constant rates for totally three times that the same experiment with MB and GAs took place, are shown.

**Figure 3:**
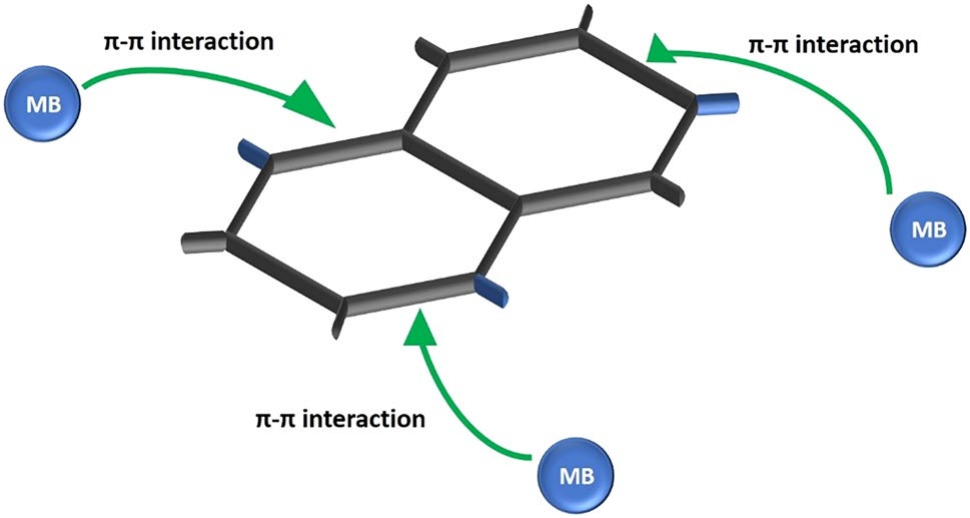
Schematic diagram showing the chemical bond between graphene aerogel and MB molecule.

The surface charge of the examined photocatalyst is significant in dye adsorption and degradation process, cationic dyes adsorb well on the surface of a material with the negative value of zeta potential and vice versa, due to the attraction of opposite charge particles ^33^. The surface charge of a sample is negative when the zeta potential is negative. Suppl. Figure 17 displays the zeta potential depending on pH. For both used GAs, the zeta potential increases for increasing pH, consequently a better performance is expected for acidic conditions. Negative values were observed for none of them, while for pH equal to 4 and 7, the FD GAs are superior to the APD ones, since their zeta potential is lower. The adsorption of MB molecule on the GAs is attributed to the fact that aerogels are structures with sufficient specific surface area and adsorption sites to remove the dye in water. More specifically for the present study, the strong π- π interaction between the benzene ring of MB and the adsorbents is the main sorption mechanism^34^ (Fig. 3), since electrostatic interactions that would also promote sorption of MB cannot be claimed (MB is a cationic dye and zeta potential was found positive).

The cleaning ability of the FD GAs was further examined for the Orange G. To determine the relation between the concentration of the aqueous solution and the absorbance, four solutions of different concentrations were tested (Supplementary Table 6). The spectra show that the absorbance is relative to the concentration of the solutions (Suppl. Figure 18). The maximum absorbance peak is observed at approximately 477 nm along with a shoulder at ~ 405 nm. This part of the spectrum is the direct result of azo-hydrazone tautomerization of the azo –N = N– bond^35^. At 330 nm and 248 nm, the spectra show characteristic bands owed to the naphthalene and benzene ring, respectively^36^.

**Figure 4:**
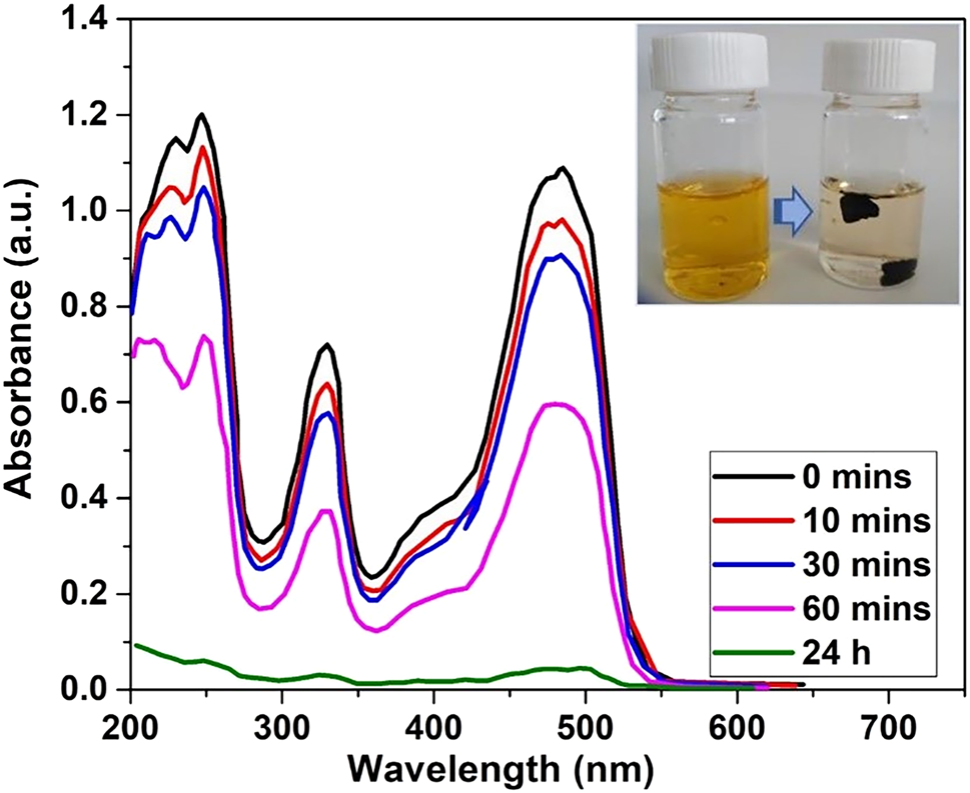
Adsorption measurements of the Orange G aqueous solution with aerogels, during the fading.

Two experiments were carried out to confirm the adsorbing and photocatalytic capacity of the GAs. Initially, an aqueous orange G solution was prepared with initial concentration 0.1 mg/ml (content of 0.0008% w/w in dye). The experiment had a duration of 24 h and the time period between the measurements was extended gradually from 10 to 30 min. Aerogels were added to the solution and under continuous stirring, fading of the colour was observed (Fig. 4).

To compare the effect of GAs in the adsorption and photolysis of the dye, another experiment was conducted, this time without light exposure of the aqueous orange G solution. A 0.002% w/w aqueous solution was prepared, 30 mL of which were added in a beaker with a GA (FD synthesis method). The spectra were obtained at first every 10 min, but later the time period was increased due to the small changes of the absorption peak (Suppl. Figure 19). . Once again, the GAs accelerate greatly the photolysis of the dye. For the photocatalytic degradation of Orange G, the constant rate has been calculated equal to 0.002135 min^-1^, while the adsorption rate of the dye in dark was equal to 25.5 mg/g.

#### Adsorption of toxic organic solvents and oils

The adsorption capacity of the synthesised GAs was determined for the following VOCs, organic solvents and oils: formaldehyde, dichloromethane, acetone, ethanol, methanol, pump oil, castor oil and silicone oil. The adsorption of the previously-mentioned pollutants was measured with the gravimetric way. A quantity of 100 mL of each selected volatile organic solvent and 50 ml of each oil were mounted in desiccators. The GAs were immersed inside the solvents and oils, and their mass was measured periodically. The adsorption capacity of the prepared graphene aerogels was determined by calculating the percentage of weight change:

$$A\%\frac{w}{w}= \frac{\left(Last\, weight\, measurement\right)-(First\, weight\, measurement)}{First\, weight\, measurement} \cdot 100$$

**Figure 5:**
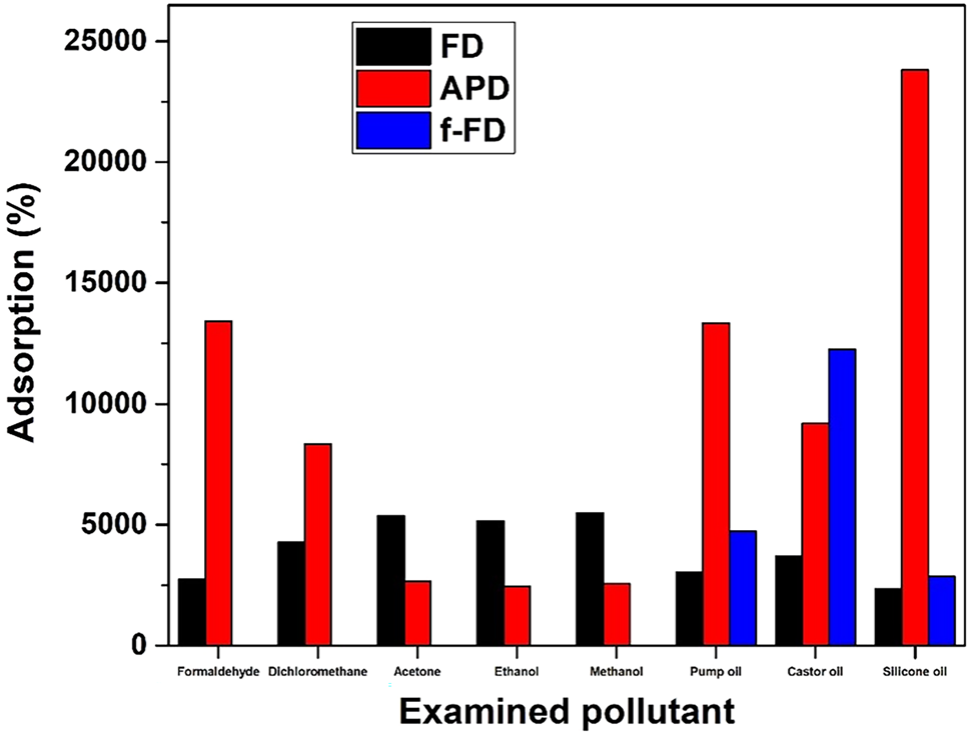
Concentrating results exhibiting the % adsorption of the examined pollutants for the prepared GAs. FD stands for freeze-drying, APD for ambient-pressure drying while f-FD represents GAs which were functionalised.

Figure 5 exhibits the concentrating results for FD, APD and functionalised FD GAs (f-FD). All synthesised GAs demonstrate high rates of adsorption for all the examined pollutants. For VOCs, the APD GAs have higher adsorption than the FD ones, while the opposite trend is depicted for the organic solvents (acetone, ethanol and methanol).APD GAs are superior for the three examined oils but the functionalisation for the FD GAs renders them better adsorbents for all the examined oils (for castor oil exceeds even the corresponding rates of the APD GAs). A remarkable performance can be obtained for the APD GAs and the adsorption of silicone oil. An adsorption rate of 23808% (meaning 238 g of adsorbed mass/1 g of adsorber) has been achieved for silicone oil showing thus the truly high adsorbing capabilities of these materials.

The as-prepared GAs show superior adsorption performance compared to other corresponding porous materials. In comparison to similar works reporting GAs, the adsorption rates for the organic solvents and examined oils in our study are either one order of magnitude higher^37–39^, or higher^40–43^or very competitive^44,45^. The adsorption rates could be enhanced if a higher concentration of the starting GO solution had been used (in our case, the concentration was equal to 2 mg/ml). The capability of these materials to adsorb a plethora of toxic organic solvents and oils renders them highly promising for applications in water purification. Furthermore, such materials have been found to adsorb better some organic solvents than commercial solutions like activated carbon, as explained following.

Various materials as pollutants removers, ranging from activated carbons to zeolites and from metal–organic frameworks (MOFs) to hyper-crosslinked polymeric resins, have been reported^46–48^. However, several drawbacks of commercially available porous materials have been reported when it comes to VOCs removal. Activated carbon cannot capture VOCs with low molecular weight like formaldehyde, and other materials may not be effective in treating polar VOCs like ammonia. The adsorption rates of commercial (Donau Carbon) activated carbon (AC) were compared to the aerogels.. AC was dried and placed inside the desiccators with the same VOCs as previously. The calculated adsorption capacity for the VOCs and the oils is presented in Supplementary Fig. 20. AC exhibits significantly lower adsorption performance than GAs, for all the examined pollutants. Comparing the commercial adsorbent to the best aerogel sample for each examined VOC, a difference of 1 (acetone, ethanol, methanol) to 3 (formaldehyde) orders of magnitude is found. An important note is that the superior VOCs adsorption by aerogels was accomplished by using samples with average weights of a few milligrams (ranging from 10 to 65 mg), while the initial weights of the commercial adsorbent were much higher, ≈2200 to 4000 mg. Also, compared to silica aerogels synthesised using cost-effective approaches which are used for solvents adsorption^49–53^, the adsorption efficiency of the GAs of this study is superior. But, the re-usability of the silica aerogels is excellent since some of them can be functional even after 100 cycles of adsorption–desorption^52^.

**Figure 6:**
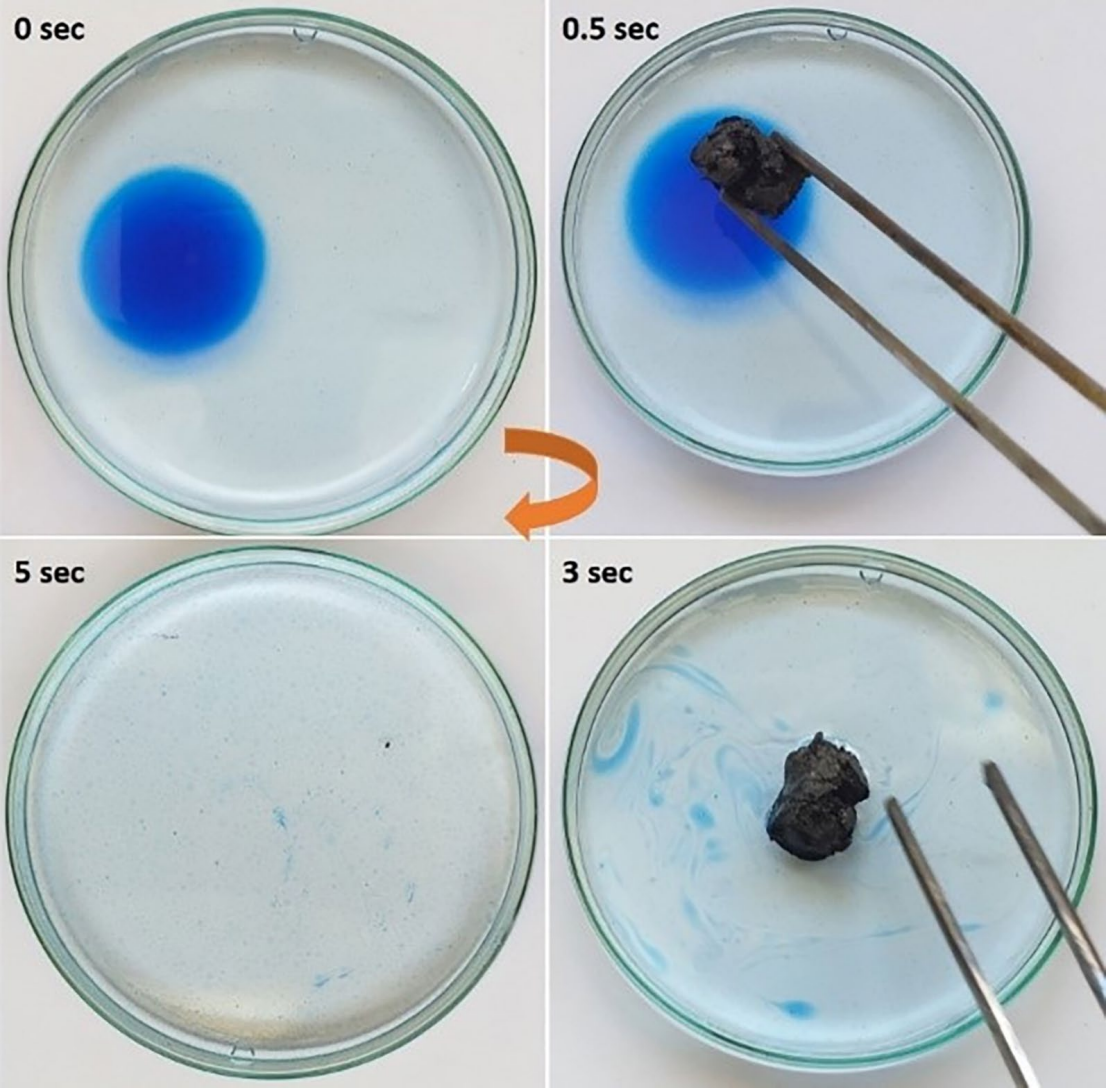
Oil adsorption test of the FD aerogel. Castor oil (stained with Oil blue N dye) floating on water was completely absorbed within 5 s.

As a result of their high porosity, mechanical stability and hydrophobicity, the GAs are ideal candidates for the sorption of oils. As depicted in Fig. 6 and Suppl. Figures 21–23, when a small piece of each examined aerogel was placed on castor oil, pump oil, silicone oil and almond oil (all strained with Oil blue N dye), the oils were immediately. The fluorinated functional groups create bonding with the remaining oxygen-functional groups of the rGO sheets and provide a hydrophobic rGO aerogel surface for a selective oil removal. In addition, the three-dimensional macroporous networks of the rGO aerogel are known to show high porosity and large accessible area^18,25^. The large surface area of the rGO aerogel combined to the hydrophobicity of fluorinated functional groups could have a synergistic action on removing oils from water.

**Figure 7:**
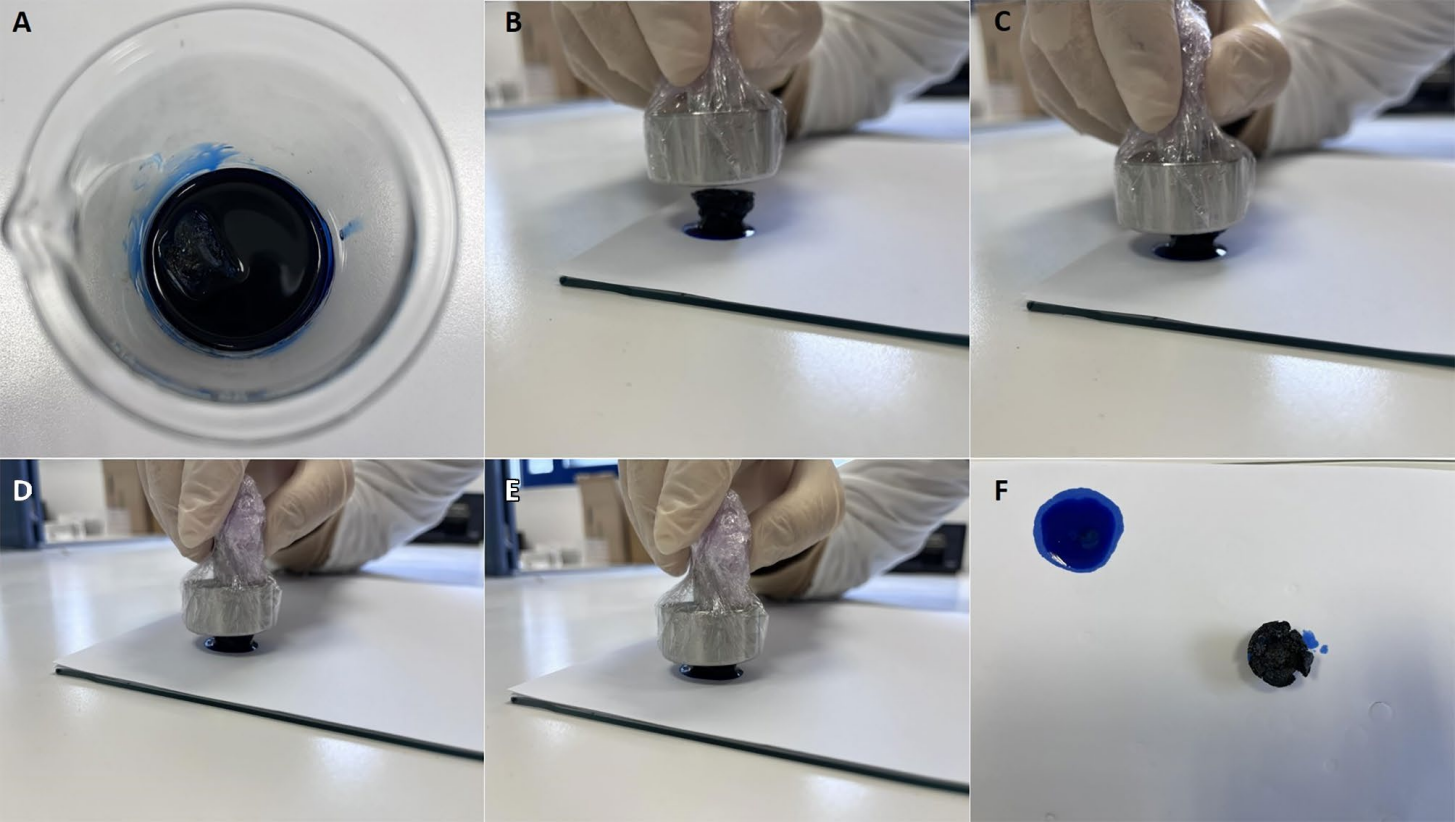
Digital images showing the process of the absorption–squeezing for oil (pump oil, dyed with Oil blue N dye) collection using the APD aerogel. In this way, the absorbed oil can be collected with high efficiency.

The solid structure and the super-elastic nature of the aerogelsresult to efficient collection of the adsorbed oil by squeezing it out (Fig. 7 and Suppl. Figure 24). For both techniques, the adsorbed oil can be collected almost fully, while, after a short period of the squeezing, the height or the volume of the samples returns to the initial state. The alternative choices of burning or evaporating the adsorbed oil are considered much harmer and less environmentally friendly than the suggested one within this study.

**Figure 8:**
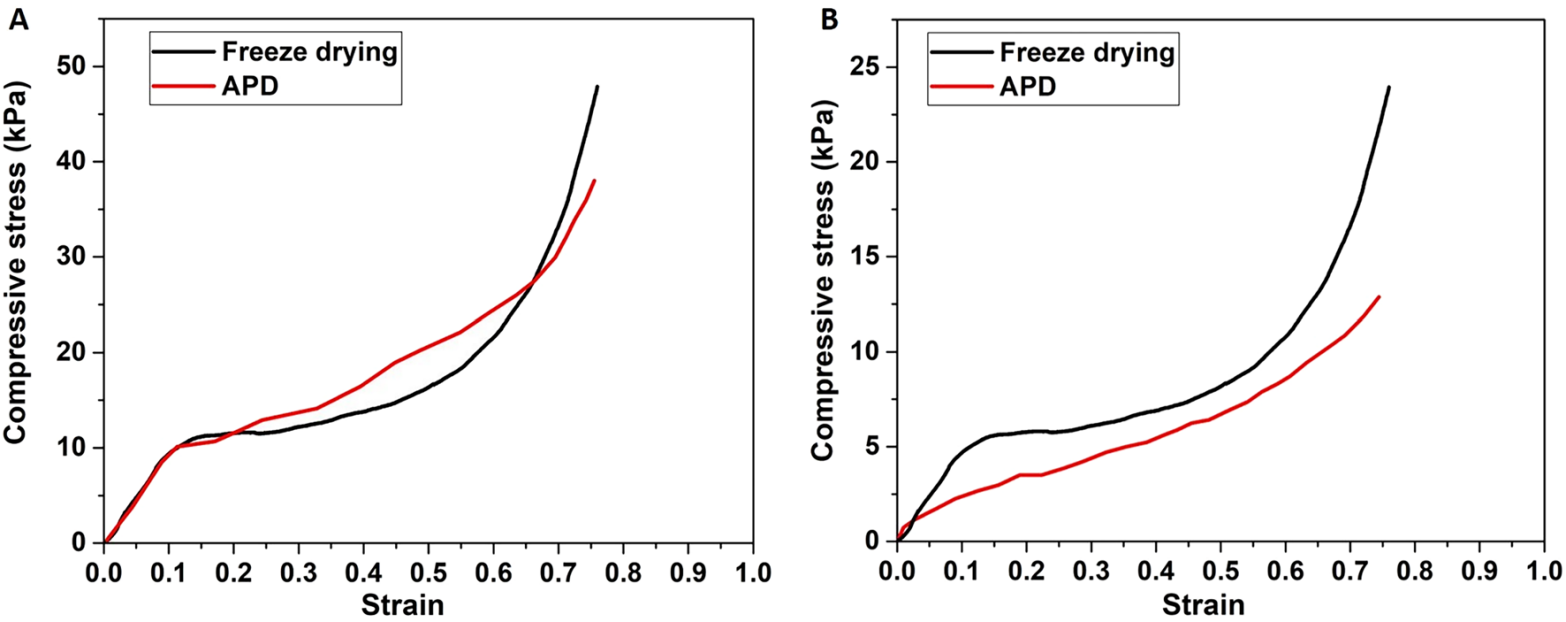
Representative stress–strain curves under compression for the as-prepared examined aerogels (**A**), and, for the aerogels during the photocatalytic degradation of dyes (**B**).

The mechanical properties under compression for both formulations were examinedin comparison to the GAs during the photocatalytic degradation of dyes (Fig. S25). In Fig. 8, representative stress–strain curves are presented, both for the as-prepared examined aerogels (**A**), and, for the aerogels during the photocatalytic degradation of dyes (**B**). All samples were compressed up to a maximum compressive strain of ≈ 75% without failure. The stress–strain curve is consisted of three discrete areas, characteristic of the compressive behaviour of aerogels. Initially a linear Hookean behaviour, which holds roughly up to 10%, is observed corresponding to the elastic bending of the cell walls^12,25^. The initial linear behaviour from which the Young’s modulus can be extracted, is sequenced by a non-linear regime of much lower modulus. During this phase, the densification of the compressed walls of the aerogels takes place, until a compressive strain of 50 − 55%. For higher compression values, the densification and the decrease of the porosity of the macro-structure result in the rapid increase of the stress. The Young’s modulus has been calculated equal to 100.00 kPa for both formulations at the initial state of the aerogels, while it turns to 49.86 kPa and 18.71 kPa for FD and APD aerogels respectively, when these are soaked with the dyed aqueous solution. The as-prepared GAs present better mechanical performance as compared to other similar structures^25,54, 55^ since for similar density, they can achieve much higher compressive stresses without failure. When the GAs were subjected to compression during the photocatalytic degradation of dyes (Fig. 8B), a similar behaviour is observed.. However, a significant decrease by half (from ≈ 48 to ≈ 24 kPa) and by one/third (from ≈ 38 to 13 kPa) in the compressive stress they can bear is observed for the FD method, and the APD methods respectively.

## Conclusions

The successful synthesis of GAs following two different routes was described within this work. In addition to the basic technique of FD, a greener technique that involves APD which uses only ascorbic acid as the reducing agent, was applied as well. A functionalisation using a fluorine-silane coupling agent for the aerogels obtained with FD is also described as an additive tool for enhancing their hydrophobicity and consequently their oil-adsorption efficiency. A full characterisation was also undertaken with SEM, Raman spectroscopy, XPS, XRD and contact angle analysis. In both classes of materials, the reduction of the GO was confirmed, whereas the C/O ratio measured by XPS for the FD samples was almost double of that obtained for the APD materials. The photo-catalytic ability of both types of aerogels was also confirmed for the MB dye with the FD technique exhibiting a superior performance. This can be attributed to the doping of the structure with phosphorus from the used reducing agent, and, to the higher degree of reduction that has sustained. High catalytic efficiency for the FD aerogels was confirmed also for the Orange G dye. Furthermore, several toxic organic solvents and oils like formaldehyde, dichloromethane, acetone, ethanol, methanol, pump oil, castor oil and silicone oil, were tested for both aerogels’ cases. Significantly high rates were achieved, while the applied functonalisation raised the rates of bare aerogels for two of the three measured oils. Finally,for five of the totally eight examined water pollutants, the APD led to graphene foams with superior adsorptions than the FD, despite their lower photocatalytic activity.

## Supplementary Information

Supplementary Information.

## Data Availability

All data generated or analysed during this study are included in this published article.
