# Correction: Graphene aerogels as efficient adsorbers of water pollutants and their effect of drying methods

**DOI:** 10.1038/s41598-026-41551-x

**Published:** 2026-03-09

**Authors:** George Gorgolis, Natalia Pastra, Maria Kotsidi, George Paterakis, Nikos Koutroumanis, Christos Tsakonas, Costas Galiotis

**Affiliations:** 1 Department of Chemical Engineering, University of Patras, 26504 Patras, Greece; 2 Foundation for Research and Technology – Hellas (FORTH/ ICE‑HT), Institute of Chemical Engineering Sciences, 26504 Patras, Greece

Correction to: *Scientific Reports* 10.1038/s41598-024-58651-1, published online 05 April 2024

The original version of this Article contained errors, where one of the authors was erroneously omitted from the author list. Specifically, the second author, Natalia Pastra, was omitted from the original author list.

As a result, the author list was corrected from “George Gorgolis^1,2^, Maria Kotsidi^1^, George Paterakis^2^, Nikos Koutroumanis^2^, Christos Tsakonas^2^ & Costas Galiotis^1,2^” to read: “George Gorgolis^1,2^, Natalia Pastra^1^, Maria Kotsidi^1^ George Paterakis^2^, Nikos Koutroumanis^2^, Christos Tsakonas^2^ & Costas Galiotis^1,2^”.

Additionally, the Author information section under subsection Contributions: “C.G. supervised and planned the research. G.G., G.P., M. K. and C.T. prepared and characterised the aerogel samples and performed the adsorption experiments. G.P., N.K. prepared the GO solutions. N.K. performed the mechanical properties measurements. G.G. and C.G. wrote the paper.”

Now reads:

“C.G. supervised and planned the research. G.G., G.P., M. K. and C.T. prepared and characterised the aerogel samples and performed the adsorption experiments. G.P., N.K. prepared the GO solutions. N.K. performed the mechanical properties measurements. G.G., C.G. wrote paper N.P participated in the synthesis of the graphene aerogels with both mentioned methods, freeze-drying and ambient pressure drying and in some part of the aerogels characterisation with Raman spectroscopy and contact angle measurements. Furthermore, she has performed the photocatalysis experiments examining the mentioned dyes (methylene blue and Orange G), while she has also proved the regeneration ability of them with drying.”

Finally, the Supplementary information was updated to include Natalia Pastra in the author list.

The Original Article has been corrected.

## Supplementary Information

Below is the link to the electronic supplementary material.

Supplementary Material 1

