# Supplementary material for: Graphene aerogels as efficient adsorbers of water pollutants and their effect of drying methods
Source: Sci Rep. 2024 Apr 5;14:8029. doi: 10.1038/s41598-024-58651-1 (PMC10997784; doi:10.1038/s41598-024-58651-1)
Supplement: Supplementary file 1 — Supplementary Information. [file 41598_2024_58651_MOESM1_ESM.docx]

Graphene aerogels as efficient adsorbers of water pollutants and their effect of drying methods

G. Gorgolis^1,2*^, N. Pastra^1^, M. Kotsidi^1^, G. Paterakis^1,2^, N. Koutroumanis^2^, C. Tsakonas^2^ and C. Galiotis^1,2*^

^1^Department of Chemical Engineering, University of Patras, Patras 26504

^2^Institute of Chemical Engineering Sciences, Foundation for Research and Technology – Hellas (FORTH/ ICE-HT), Patras 26504, Greece

[galiotis@chemeng.upatras.gr](mailto:galiotis@chemeng.upatras.gr), [c.galiotis@iceht.forth.gr](mailto:c.galiotis@iceht.forth.gr)[, ggorgolis@iceht.forth.gr](mailto:%2Cggorgolis@iceht.forth.gr)

Graphene Oxide preparation

Graphene oxide (GO) is synthesised from natural graphite flakes (NGS Naturgraphit GmbH, Germany) by a two-steps oxidation process, based on Kovtyukhova, N.I., et al. method [1] (which is a modification of Hummer method), namely, a pre-oxidation step at the beginning, followed by the final oxidation step where GO flakes are collected in an aqueous dispersion. At the first step, 10g. of natural graphite flakes are added in 75 ml concentrated sulfuric acid (H2SO4, 96%) in a flask. The flask is placed in a bath and heated at 80°C. After that, 5g. of potassium persulfate (K_2_S_2_O_8_) and 5g. of phosphorus pentoxide (P_2_O_5_) are added in the solution. The mixture is stirred for 1 h at this temperature and then is allowed to cool at room temperature in a period of 5 h. At the end of this process a dark blue solution is produced. The reaction is terminated by carefully adding deionized water (DW), followed by several steps of vacuum filtration with DW, until pH reaches pH of DW. Finally, the pre-oxidized graphite was dried overnight under ambient conditions. The oxidized graphite is then subjected to oxidation by modified Hummer’s method, where the powder is stirred continuously into a flask with 220 ml of H_2_SO_4_, 96%, in a water bath. Gradually, 26.7 g. of potassium permanganate (KMnO_4_) were added in the mixture, making sure that the temperature of the system is not allowed to reach 20°C. Thereafter, the mixture is heated to 40°C for 2 h, where 450 ml of DW are added carefully. After 15 min the reaction was terminated by adding 1.35 liters of DW and 22 ml of hydrogen peroxide (H_2_O_2_, 30%). The bright yellow mixture is filtered and washed with 1:10 HCl solution in order to remove most of the metal ions. The solid product of this process is redispersed in DW, and subjected to dialysis until the pH becomes the same with the pH of DW. Finally, single and few layers of GO are collected by a combination of ultra-sonication and centrifugation steps.

Freeze-drying method

For obtaining GAs via the freeze-drying process (**Supplementary Figure 1**), the following steps were performed: firstly, a GO aqueous solution of 2 mg/mL concentration in a cylindrical vial was used and then, hypophosphorous acid (H_3_PO_2_) and iodine (I_2_) as the reducing agents were added. Afterwards, the graphene hydrogels in the oven (80°C, 8 hours) were formed, rinsed with distilled water until neutral pH is reached, and, freeze-dried for 48-72 hours to obtain the graphene aerogel. Based on the proposed self-assembly mechanism [2] of the graphene sheets, at first H_3_PO_2_ is combined with I_2_ and creates hydriodic acid that acts as the reducing agent and is responsible for the pH decrease of the medium (pH<1). The decreased value of pH results in diminished

electrostatic repulsions, which then aids the formation of GO agglomerates. Then, the oxygen- containing functional groups on the GO surface are reduced from hydriodic acid and produce chemically converted graphene.

Surface modification

In order to increase the hydrophobicity of the aerogels and to enhance their capacity to adsorb oil from an aqueous solution, the surface of the materials was modified using a fluorine-silane coupling agent. The functionalization process was as follows: 2.4 mg of trichloro(1H,1H, 2H, 2H- heptadecafluorodecyl)silane in 60% toluene from Sigma- Aldrich were used, and, the GAs were soaked for approximately 24 hours in an airtight vessel at room temperature. Then, the GAs were rinsed by consequently soaking each of them for a few seconds in three beaks of 3D water, and, dried overnight at 40°C. Firstly, the fluorine-silane agent derives -Si-OH groups, which are considered particularly reactive toward protic groups and react with the remaining hydroxyl group of C-OH of reduced graphene oxide sheets. The reactive silane groups establish covalent bonds with -OH groups, leading to the formation of Si-O-C bonds.

From the **Supplementary Figures 2** and **3**, it can be deduced that when using fluorosilane coupling agent for the surface of the material, the expected reaction has indeed occurred. More specifically, after the functionalisation, the XRD spectrum shows a broad peak at 2θ = 26° which corresponds to the (002) plane of graphite structure and indicates the successful reduction of the graphene oxide. Also, some other more sharp and intense peaks are shown and are attributed to fluorine and silicon [3][4][5] of the used chemical for the functionalisation of the graphene aerogel. X-ray photoelectron spectroscopy (XPS) was used to monitor the changes in surface chemistry of rGO aerogel after functionalization. The XPS survey scan is an effective way to determine the presence of C, F and Si elements on the surface of a material. General survey spectrum and spectra for fluorine and silicon are shown in **Supplementary Figure 3A–C** for a functionalised rGO aerogel, respectively. The oxygen content of a functionalised rGO aerogel is higher than that of the pristine rGO aerogel, because the fluorine silane is linked to the rGO aerogel surface by the C– O–Si bonds resulting in more oxygen left after the surface modification.


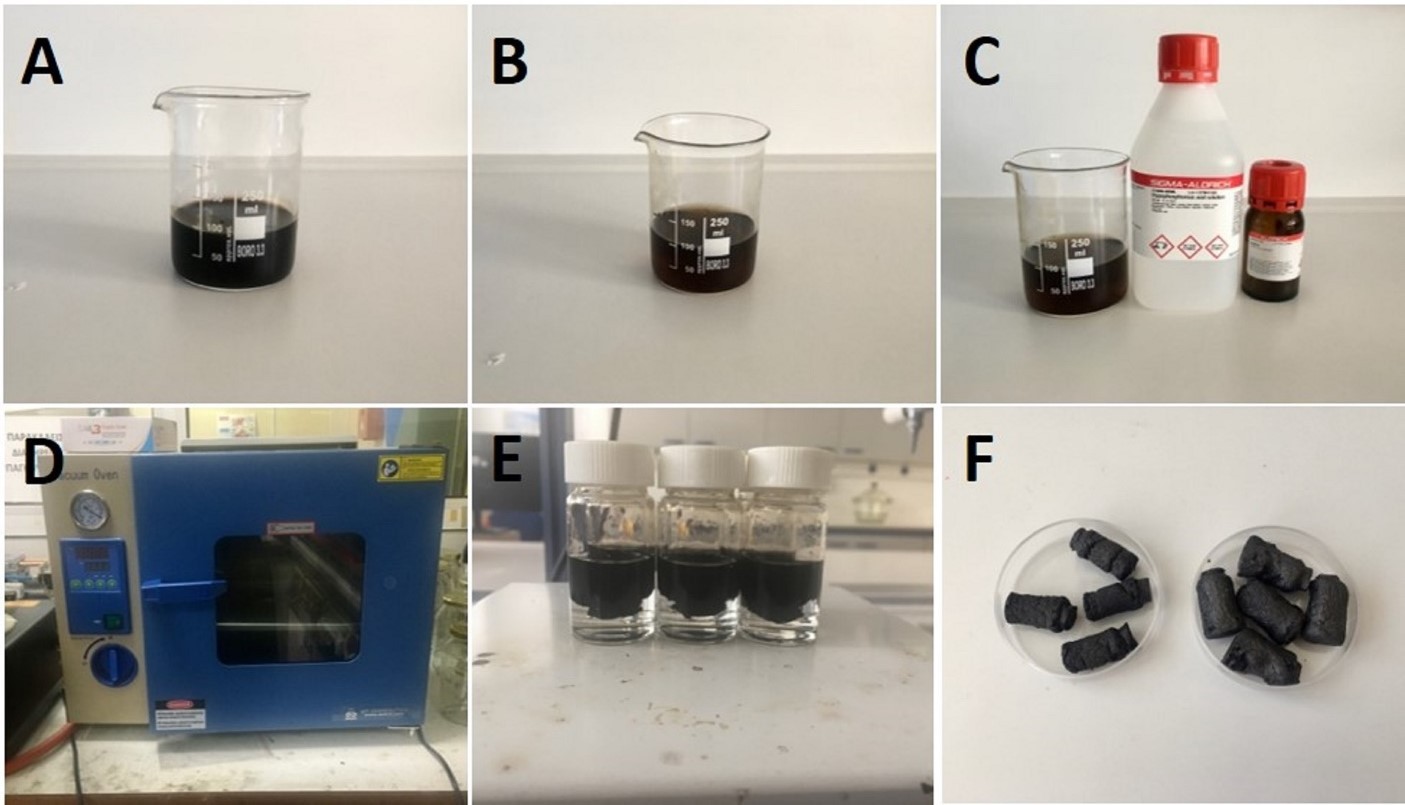


***Supplementary Figure 1****: Schematic representation of the experimental process. A) GO aqueous solution of 3.2 mg/ml, B) GO dilution to 2 mg/ml – Sonication for 3-4 minutes, C) Addition of H_3_PO_2_ and I_2_, D) Self-assembly and reduction at 80°C for 8 h, E) Graphene hydrogels and F) Graphene aerogels.*

*
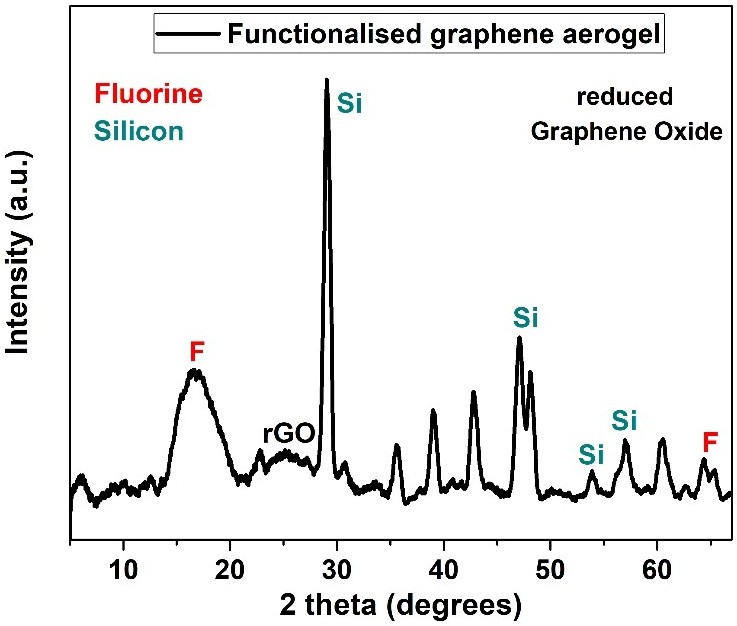
*

***Supplementary Figure 2****: XRD spectrum for a functionalised rGO aerogel.*


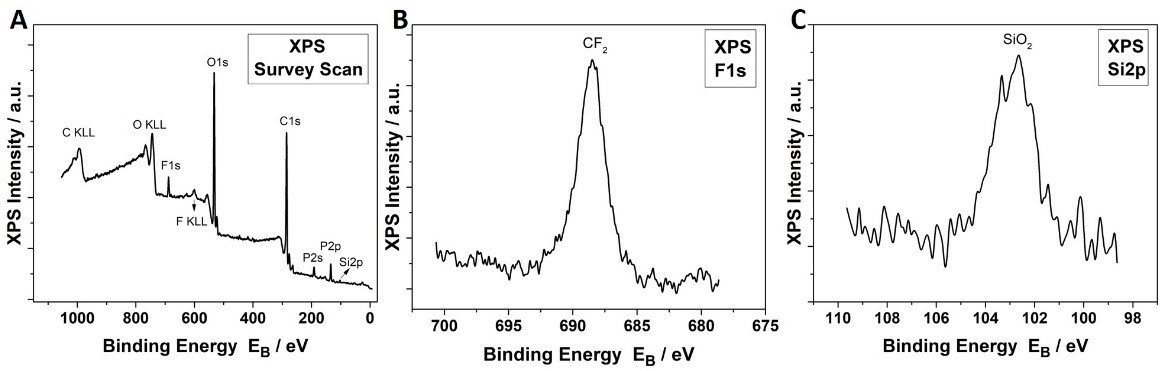


***Supplementary Figure 3****: XPS survey scan for the functionalised aerogel. Full range scan (A) after surface modification, (B) F1s binding energies after surface modification and (C) Si2p binding energies after surface modification.*

Ambient-pressure drying method

The alternative of APD suggests significantly less energy consumption and a more facile approach that could potentially be scaled-up. The main characteristics of the method is the chemical reduction of GO and the fact that it does not require a special apparatus (like in freeze- and supercritical-drying). The fabrication process includes: a GO aqueous dispersion of 2 mg/mL concentration, 80 mg of L-Ascorbic Acid (L-AA) for 10 mL of GO, stirring so as to achieve uniform mixing, the formation of a partially reduced graphene hydrogel via heating in the oven (95°C, 1 hour), cooling of the hydrogel at room temperature, freezing at -18°C for at least 24 hours, thawing of the hydrogel at room temperature, formation of the reduced graphene hydrogel via heating in the oven (95°C, 5 hour), rinsing of the graphene aerogel in order to remove soluble pieces and to reach a neutral pH, and finally drying in the oven at 60°C for 24h to obtain the graphene aerogel. **In Supplementary Figure 4**, some large aerogels prepared with the APD method are shown.


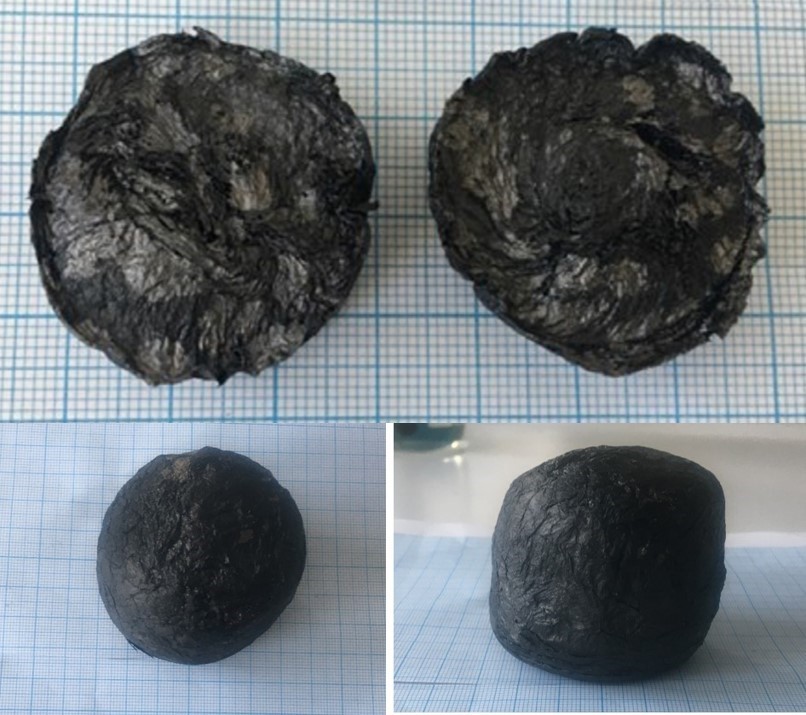


***Supplementary Figure 4****: Fully reduced graphene aerogels obtained with the ambient-pressure drying method.*

*
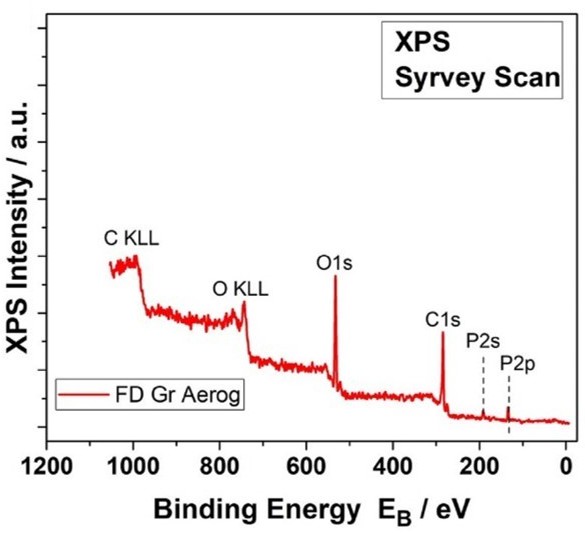
*

***Supplementary Figure 5****: XPS survey scan of the FD sample.*

***Supplementary Table 1****: C1s component concentration derived from the C1s peak deconvolution (****fig. 1D****) and relative atomic ratio C:O*

| **Component** | **[%]** |
| --- | --- |
| C-C sp^2^ | 69.02 ± 6.93 |
| C-C sp^3^ | 3.90 ± 4.70 |
| C-O(H) | 14.29 ± 5.90 |
| C=O | 5.66 ± 4.30 |
| COOH | 5.59 ± 1.45 |
| pi-pi^*^ | 1.54 ± 0.26 |

***Supplementary Table 2****: Exemplary values of C:O ratios for graphene aerogels*

| **C/O Ratio** | **Reference** |
| --- | --- |
| 9.59 | [6] |
| 9.08 | [7] |
| 4.1 | [8] |
| 7.58 | [9] |
| 6.98 | [10] |

*
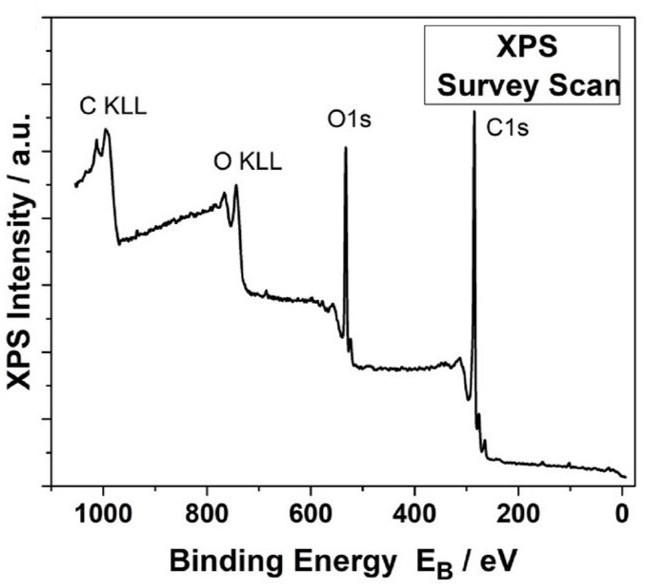
*

***Supplementary Figure 6****: XPS survey scan of the APD sample.*


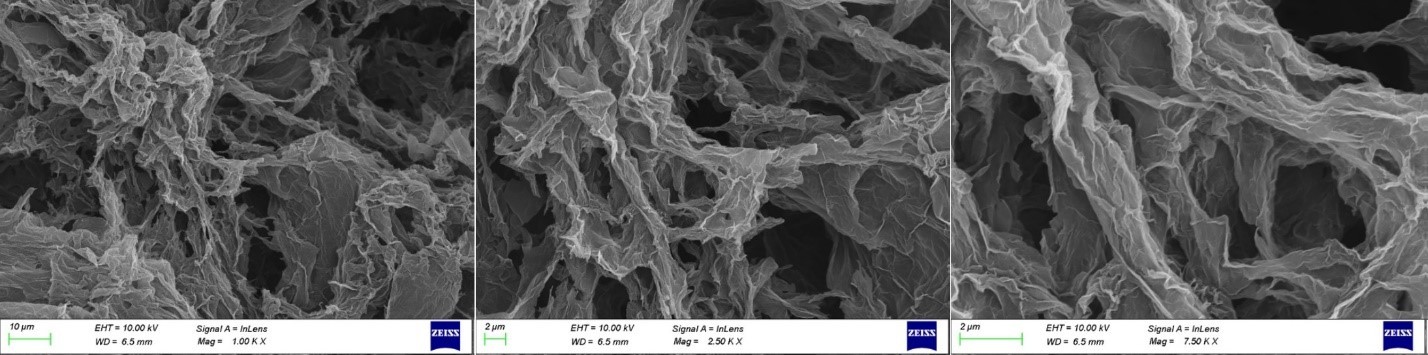


***Supplementary Figure 7****: SEM images at 10 μm and 2 μm for graphene aerogels prepared with*

*FD method.*

*
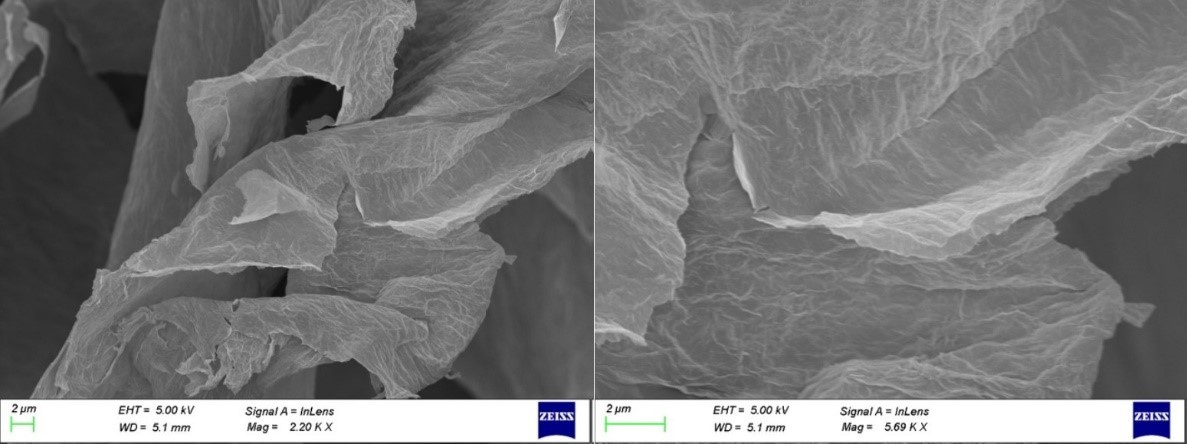
*

***Supplementary Figure 8****: SEM images at 2 μm for graphene aerogels prepared with APD method.*

*
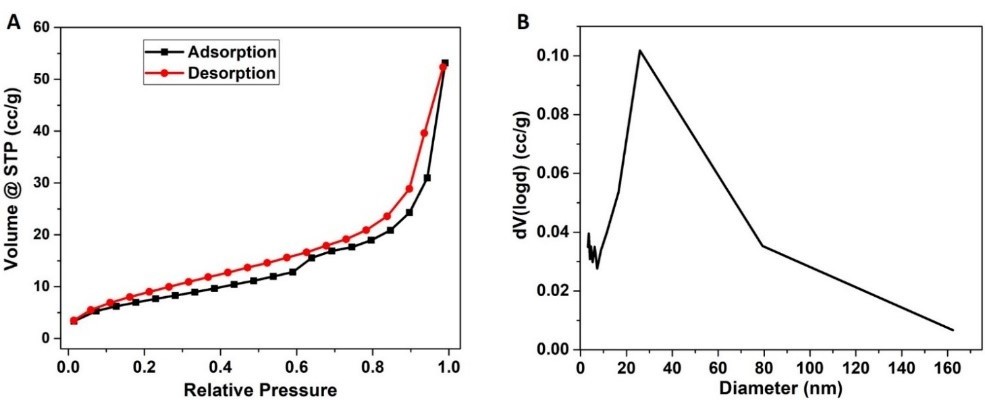
*

***Supplementary Figure 9****: A) Pore characterisation of the examined FD aerogel. The nitrogen adsorption–desorption isotherm of the aerogel is shown. The BET surface area and nitrogen*

*adsorption–desorption isotherms were measured at using the analysis program of the micropore physio-/chemisorption analyser. B) Barrett-Joyner-Halenda (BJH) pore size distribution curve of the examined aerogel.*

***Supplementary Table 3****: Pores characteristics (volume and surface area) for corresponding pore diameters of the FD aerogel.*

| **Diameter (nm)** | **Pore Volume (cm^3^/g)** | **Pore Surface Area (m^2^/g)** |
| --- | --- | --- |
| 3.1827 | 0.00173 | 2.1764 |
| 3.5721 | 0.00374 | 4.4207 |
| 4.0274 | 0.00538 | 6.0537 |
| 4.572 | 0.00737 | 7.7917 |
| 5.2425 | 0.00922 | 9.2021 |
| 6.0928 | 0.0116 | 10.77 |
| 7.2249 | 0.01379 | 11.978 |
| 8.8378 | 0.01698 | 13.422 |
| 11.417 | 0.02192 | 15.153 |
| 16.5342 | 0.0319 | 17.568 |
| 25.8787 | 0.05223 | 20.711 |
| 79.3465 | 0.0735 | 21.783 |
| 162.3338 | 0.07478 | 21.814 |

*
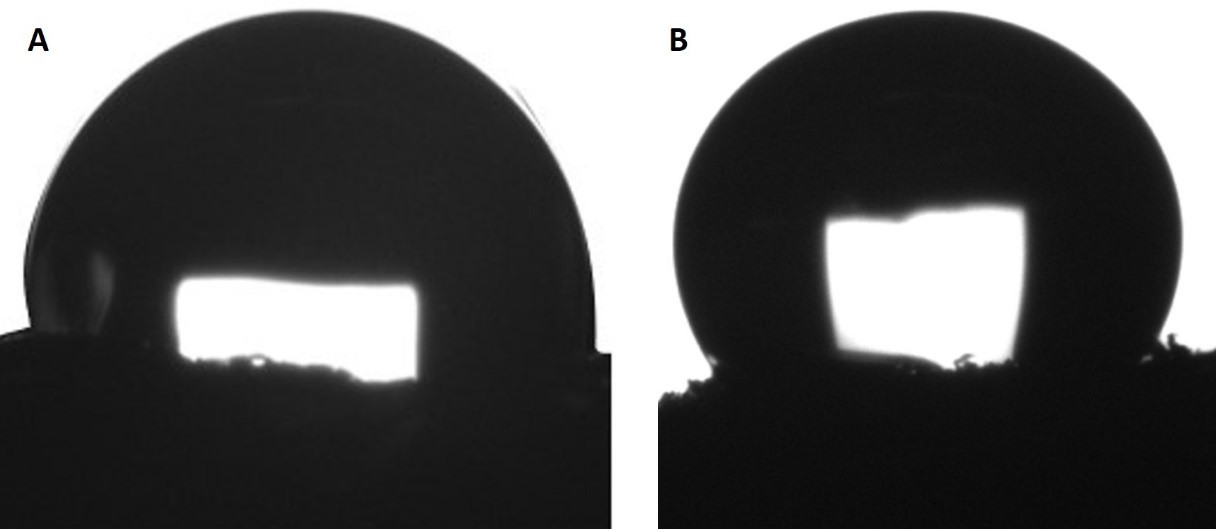
*

***Supplementary Figure 10****: Water contact angle images. A) stands for the FD aerogel and the contact angle is equal to 90.9°, while B) stands for the functionalised aerogel with a contact angle equal to 118.3°.*


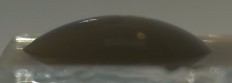


***Supplementary Figure 11****: Water contact angle measurement on glass substrate coated with Graphene Oxide (GO) film, exhibiting its hydrophilic nature. The water contact angle is equal to*

*36.0 ± 0.2°* [11]*.*

*
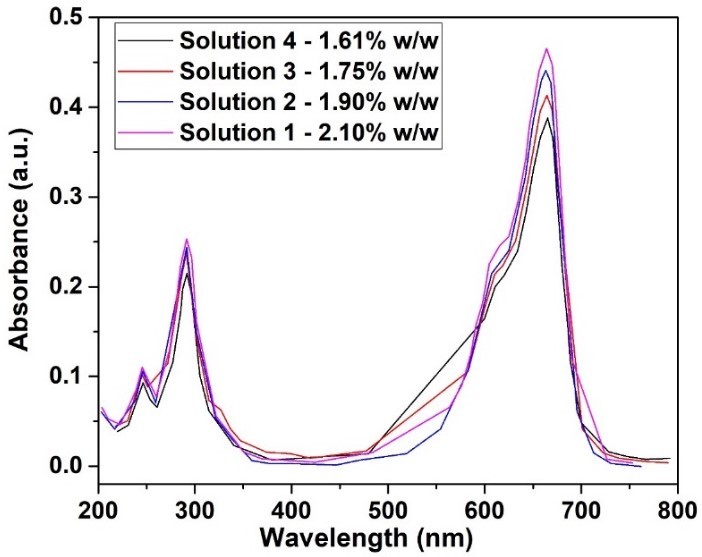
*

***Supplementary Figure 12****: UV-Vis’s spectrum of methylene blue of four different concentrations of aqueous solutions.*

**Supplementary Table 4**: Composition of aqueous MB solutions and the corresponding absorbance peaks of the UV-Vis spectra

| **Solution** | **Methylene blue (mg)** | **Distilled water (mg)** | **% content w/w** | **Absorbance peak (a.u.)** | **Wavelength (nm)** | **Absorbance peak (a.u.)** | **Wavelength (nm)** |
| --- | --- | --- | --- | --- | --- | --- | --- |
| 1 | 2.1 | 100 | 2.10 | 0.25 | 292 | 0.47 | 664 |
| 2 | 2.1 | 110 | 1.90 | 0.245 | 292 | 0.43 | 666 |
| 3 | 2.1 | 120 | 1.75 | 0.242 | 292 | 0.41 | 666 |
| 4 | 2.1 | 130 | 1.61 | 0.21 | 292 | 0.39 | 664 |


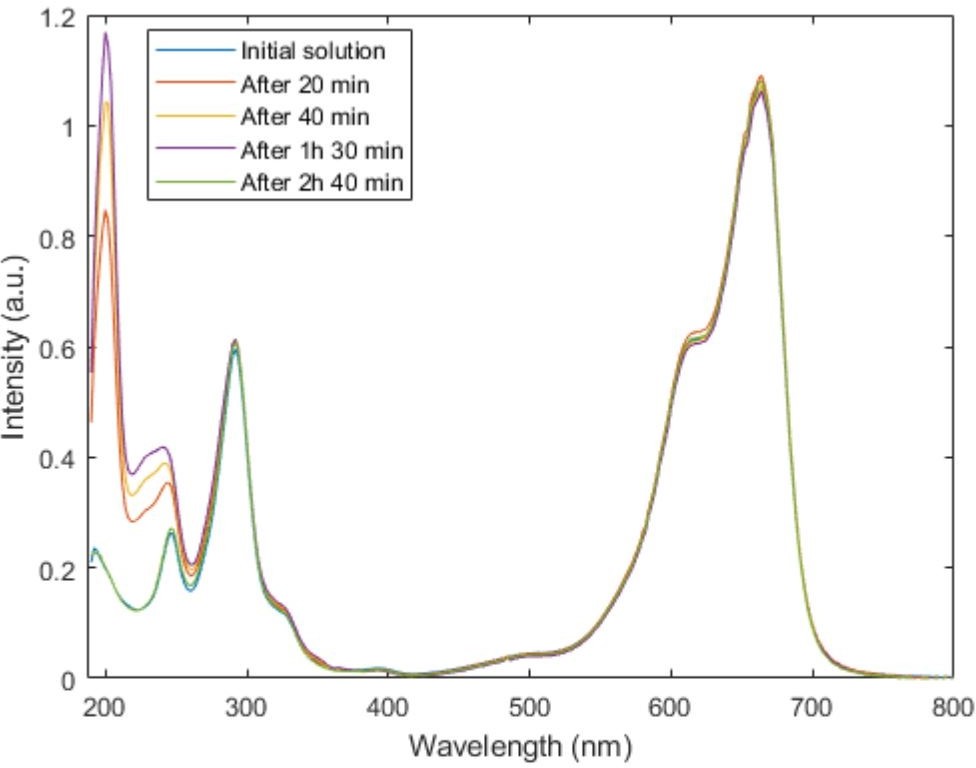


***Supplementary Figure 13****: UV-Vis’s spectra of aqueous MB solution with graphene aerogel (FD*

*synthesis method) without light exposure.*

*
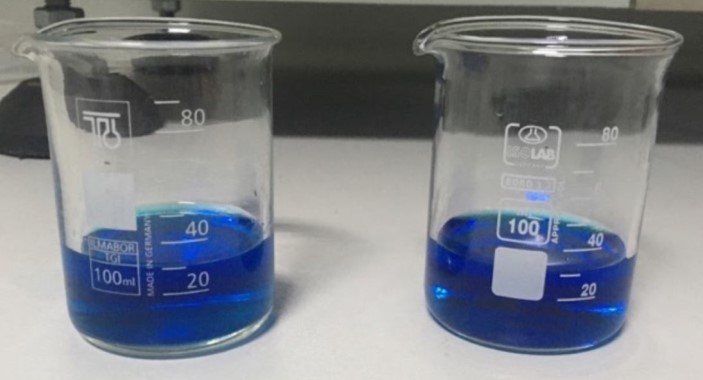
*

***Supplementary Figure 14****: Initial MB solution (right), and, the solution at the end of the experiment with GA and without light (left).*


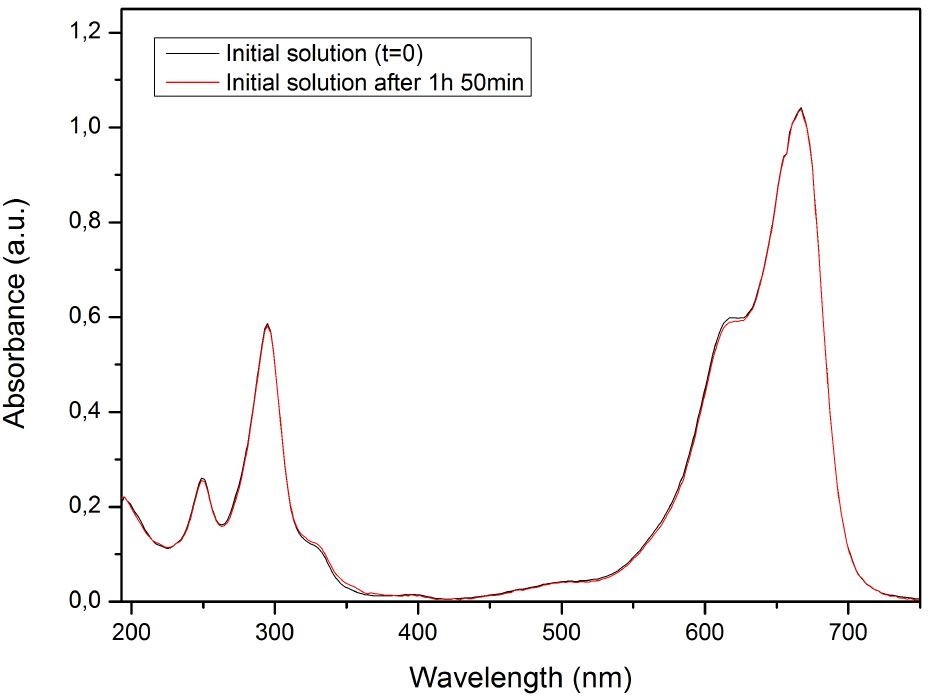


***Supplementary Figure 15****: UV-Vis’s spectra of aqueous MB solution without graphene aerogel exposed to light.*

*
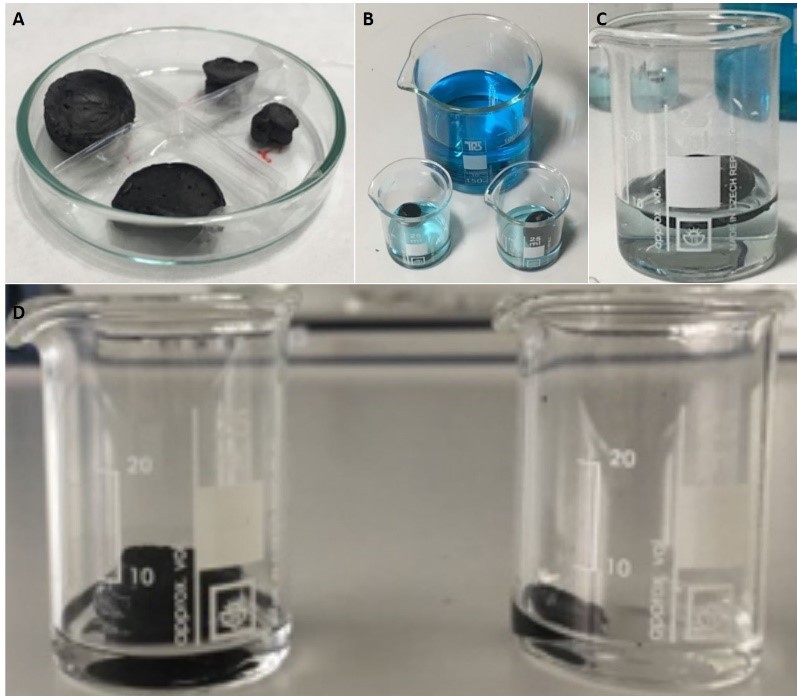
*

***Supplementary Figure 16****: A) The used graphene aerogels (FD synthesis method), B) Initial solution (centre) and the discoloured solutions containing GAs (left and right), C) Discoloured aqueous MB solution with GA at the end of the experiment, and, D) Discoloured aqueous MB solutions with GAs after two days.*

**Supplementary Table 5**: Photodegradation of methylene blue constant rates for GAs prepared by both methods before and after their regeneration, in min^-1^.

| **Number of use** | **FD GAs** | **APD GAs** |
| --- | --- | --- |
| 1 | 0.013276 | 0.003020 |
| 2 | 0.013253 | 0.003002 |
| 3 | 0.013156 | 0.002788 |


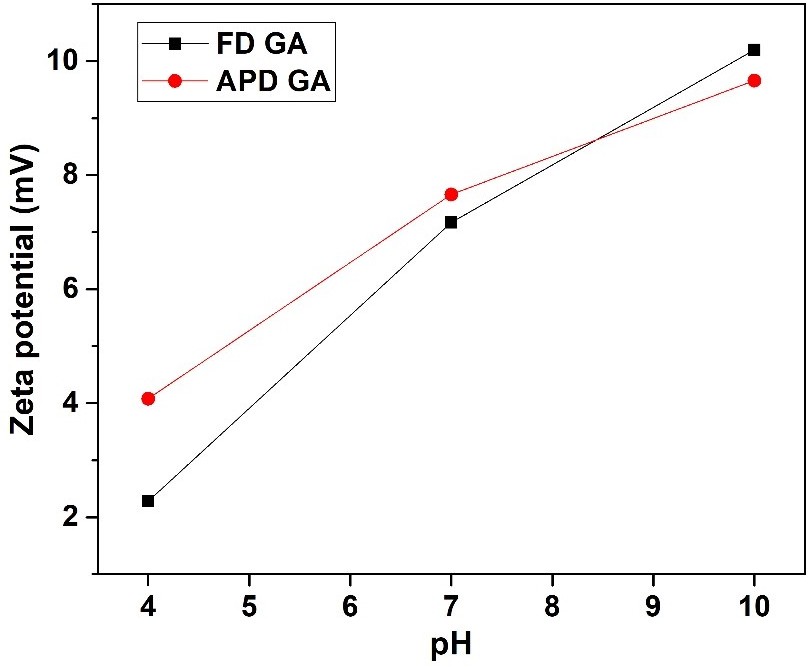


***Supplementary Figure 17****: Zeta potential measurements of the two GA formulations for three different pH values.*

**Supplementary Table 6**: Composition of aqueous Orange G solutions and the corresponding absorbance peaks of the UV-Vis spectra

| **Solution** | **Orange G (mg)** | **Distilled Water**  **(mg)** | **%**  **content w/w** | **Absorbance peak (a.u.)** | **Wavelength (nm)** | **Absorbance peak (a.u.)** | **Wavelength (nm)** | **Absorbance peak (a.u.)** | **Wavelength (nm)** |
| --- | --- | --- | --- | --- | --- | --- | --- | --- | --- |
| 1 | 1.1 | 40.24 | 2.73 | 0.41 | 478 | 0.25 | 330 | 0.50 | 248 |
| 2 | 1.1 | 50.24 | 2.18 | 0.31 | 478 | 0.21 | 330 | 0.40 | 248 |
| 3 | 1.1 | 60.24 | 1.82 | 0.26 | 476 | 0.17 | 330 | 0.33 | 248 |
| 4 | 1.1 | 70.24 | 1.56 | 0.22 | 476 | 0.15 | 330 | 0.27 | 248 |


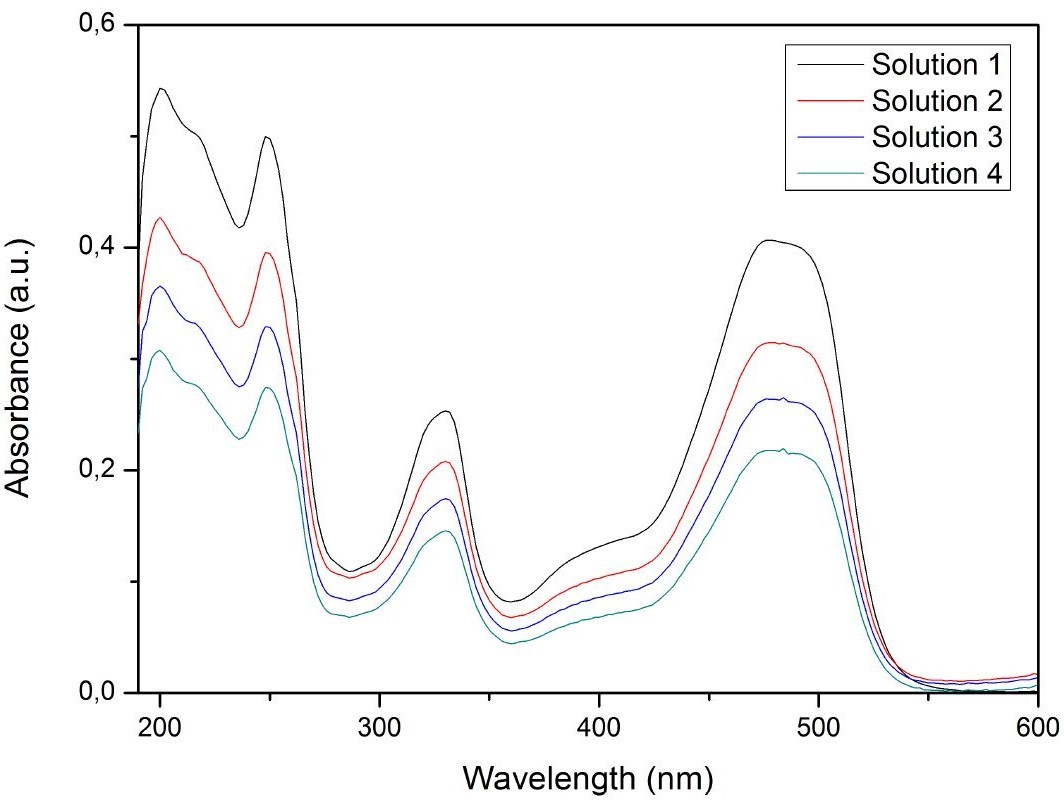


***Supplementary Figure 18****: UV-Vis’s spectrum of orange G of four aqueous solutions with different concentrations.*

*
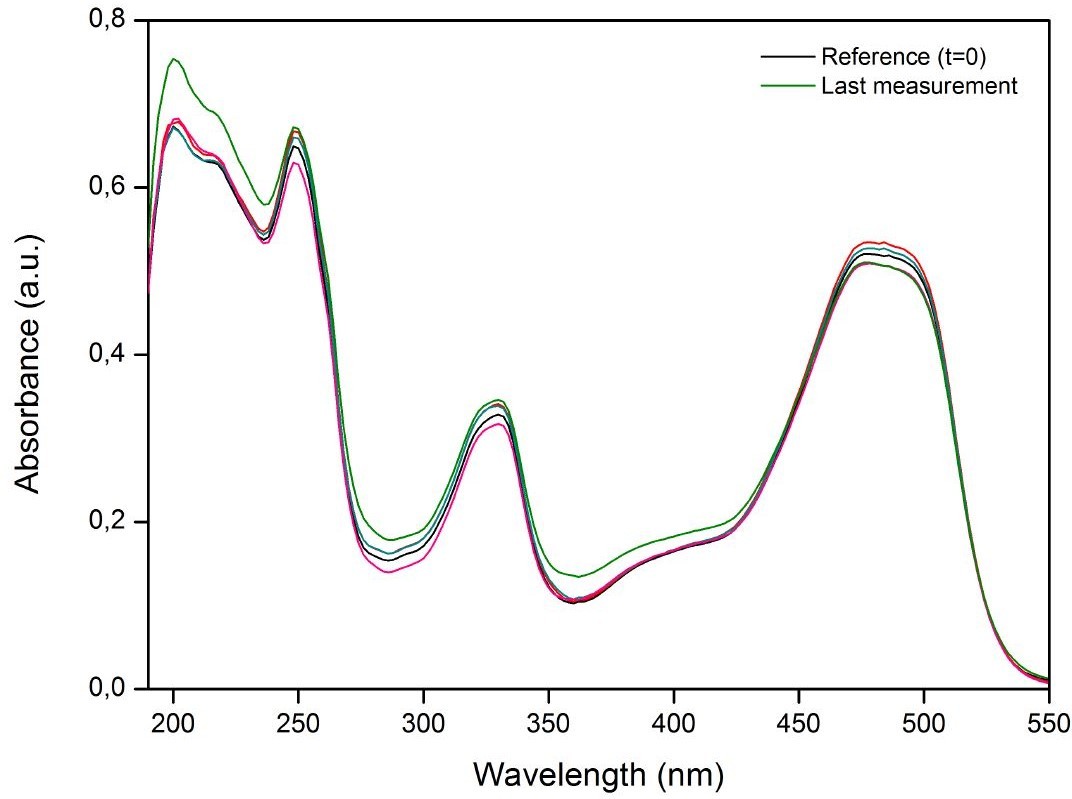
*

***Supplementary Figure 19****: UV-Vis’s spectrum of orange G aqueous solution with graphene aerogel (FD synthesis method) without light exposure.*


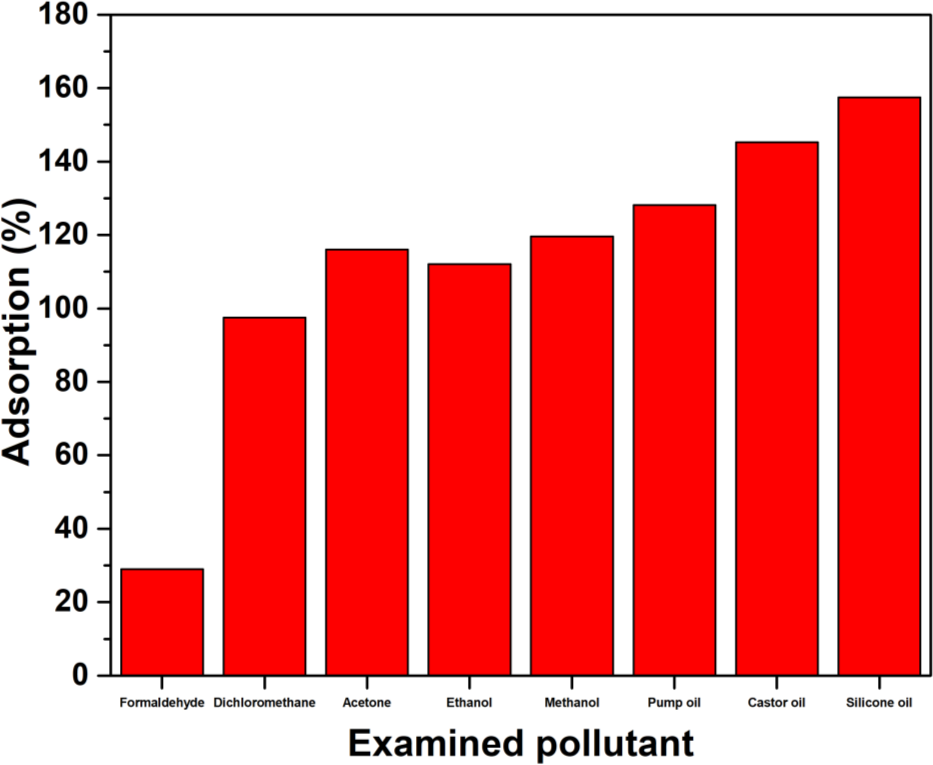


***Supplementary Figure 20****: Concentrating results exhibiting the % adsorption of the examined pollutants for the commercial activated carbon.*

*
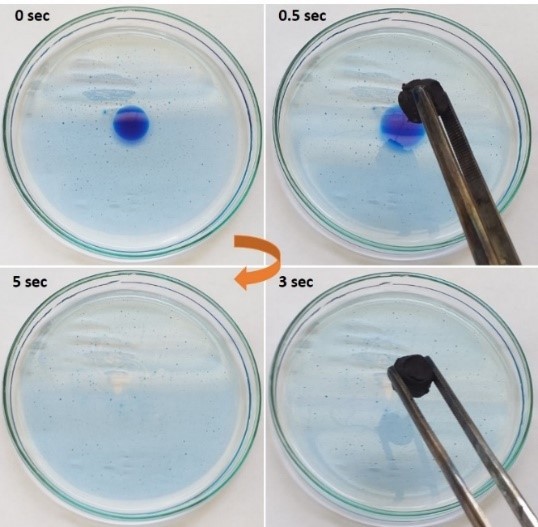
*

***Supplementary Figure 21****: Oil adsorption test of the f-FD aerogel. Pump oil (stained with Oil blue N dye) floating on water was completely absorbed within 5 s.*


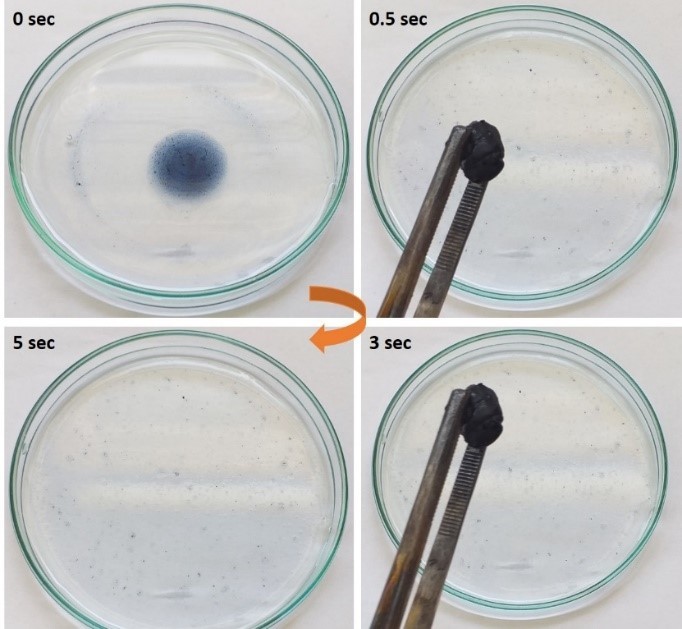


***Supplementary Figure 22****: Oil adsorption test of the FD aerogel. Silicone oil (stained with Oil blue N dye) floating on water was completely absorbed within 5 s.*

*
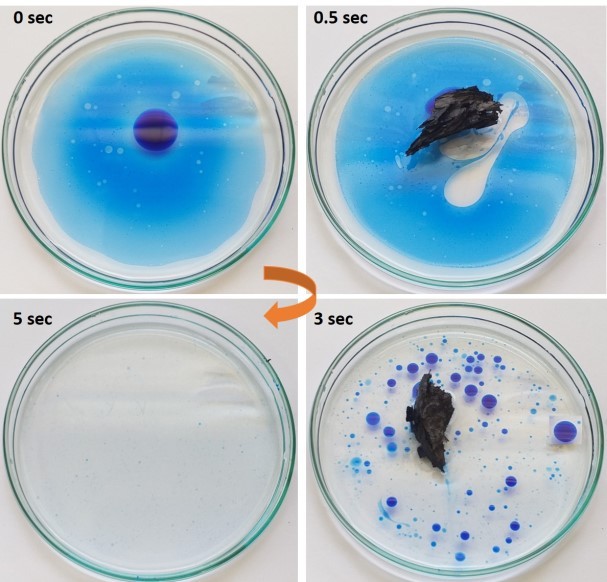
*

***Supplementary Figure 23****: Oil adsorption test of the APD aerogel. Almond oil (stained with Oil blue N dye) floating on water was completely absorbed within 5 s.*


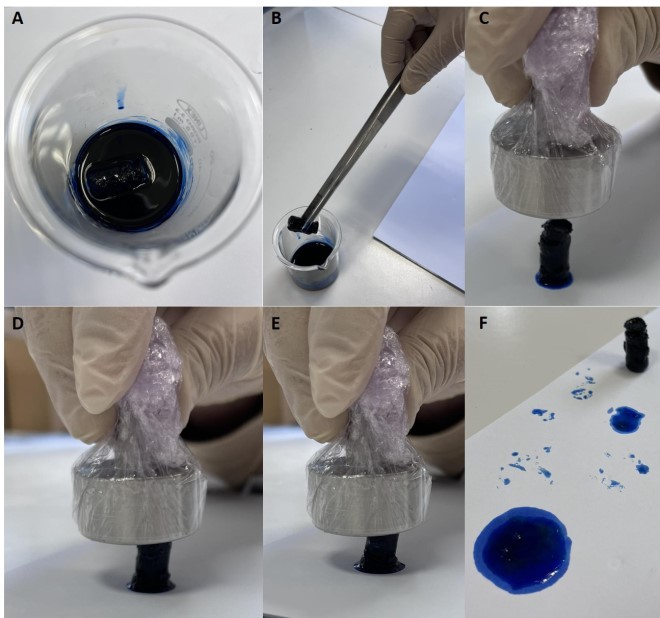


***Supplementary Figure 24****: Digital images showing the process of the absorption–squeezing for oil (pump oil, dyed with Oil blue N dye) collection using the FD aerogel. In this way, the absorbed oil can be collected with high efficiency.*

*
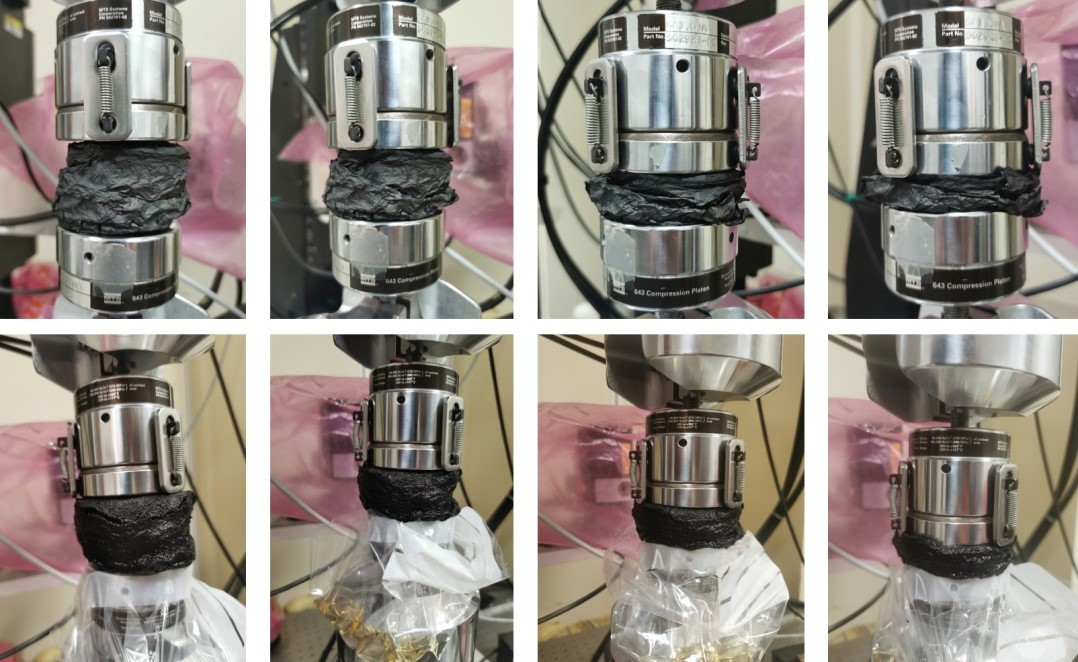
*

***Supplementary Figure 25****: Snapshots during the mechanical compression of the examined aerogels, in a dried state (upper row), and, during the photocatalytic degradation of methylene blue.*

**References**

1. N. I. Kovtyukhova, P. J. Ollivier, B. R. Martin, T. E. Mallouk, E. V. Buzaneva, and A. D. Gorchinskiy, “Layer-by-layer assembly of ultrathin composite films from micron-sized graphite oxide sheets and polycations,” *Chem. Mater.*, vol. 11, no. 3, pp. 771–778, 1999, doi: 10.1021/cm981085u.
2. H. D. Pham *et al.*, “Synthesis of the chemically converted graphene xerogel with superior electrical conductivity,” *Chem. Commun.*, vol. 47, no. 34, pp. 9672–9674, 2011, doi: 10.1039/c1cc13329b.
3. P. Gong *et al.*, “Photochemical synthesis of fluorinated graphene via a simultaneous fluorination and reduction route,” *RSC Adv.*, vol. 3, no. 18, pp. 6327–6330, 2013, doi: 10.1039/c3ra22029j.
4. M. Yoshio, H. Wang, K. Fukuda, T. Umeno, N. Dimov, and Z. Ogumi, “Carbon-Coated Si as a Lithium-Ion Battery Anode Material,” *J. Electrochem. Soc.*, vol. 149, no. 12, p. A1598, 2002, doi: 10.1149/1.1518988.
5. S. Patra, P. Mitra, and S. K. Pradhan, “Preparation of nanodimensional CdS by chemical dipping technique and their characterization,” *Mater. Res.*, vol. 14, no. 1, pp. 17–20, 2011, doi: 10.1590/S1516-14392011005000015.
6. J. Y. Hong, E. H. Sohn, S. Park, and H. S. Park, “Highly-efficient and recyclable oil absorbing performance of functionalized graphene aerogel,” *Chem. Eng. J.*, vol. 269, pp. 229–235, 2015, doi: 10.1016/j.cej.2015.01.066.
7. H. Yang, T. Zhang, M. Jiang, Y. Duan, and J. Zhang, “Ambient pressure dried graphene aerogels with superelasticity and multifunctionality,” *J. Mater. Chem. A*, vol. 3, no. 38,

pp. 19268–19272, 2015, doi: 10.1039/c5ta06452j.

1. T. Liu, M. Huang, X. Li, C. Wang, C. X. Gui, and Z. Z. Yu, “Highly compressible anisotropic graphene aerogels fabricated by directional freezing for efficient absorption of organic liquids,” *Carbon N. Y.*, vol. 100, pp. 456–464, 2016, doi: 10.1016/j.carbon.2016.01.038.
2. L. Xu *et al.*, “Superhydrophobic and superoleophilic graphene aerogel prepared by facile chemical reduction,” *J. Mater. Chem. A*, vol. 3, no. 14, pp. 7498–7504, 2015, doi: 10.1039/c5ta00383k.
3. F. Wang *et al.*, “Facile synthesis of ultra-light graphene aerogels with super absorption capability for organic solvents and strain-sensitive electrical conductivity,” *Chem. Eng. J.*, vol. 320, pp. 539–548, 2017, doi: 10.1016/j.cej.2017.03.082.
4. S. Matsalis, G. Paterakis, N. Koutroumanis, G. Anagnostopoulos, and C. Galiotis, “Fabrication and performance of capacitive humidity and strain sensors that incorporate 3D-printed nanocomposite electrodes,” *Sensors Int.*, vol. 5, no. October 2023, 2024, doi: 10.1016/j.sintl.2023.100272.
